# Integrated multiple analytes and semi-mechanistic population pharmacokinetic model of tusamitamab ravtansine, a DM4 anti-CEACAM5 antibody-drug conjugate

**DOI:** 10.1007/s10928-021-09799-0

**Published:** 2022-02-15

**Authors:** Clemence Pouzin, Leonid Gibiansky, Nathalie Fagniez, Mustapha Chadjaa, Michel Tod, Laurent Nguyen

**Affiliations:** 1 Sanofi R&D, Pharmacokinetics Dynamics and Metabolism Department, 1 Avenue Pierre Brossolette, Chilly-Mazarin, 91380 Paris, France; 2 Oncology department EMR3738, PKPD modelling unit, University of Claude Bernard Lyon 1, Lyon, France; 3 QuantPharm LLC, North Potomac, Maryland USA; 4 Sanofi, Clinical Research, Vitry-sur-seine, Paris, France

**Keywords:** Antibody-drug conjugate, CEACAM5, DM4, Drug-to-antibody-ratio, Pharmacokinetics model, Semi-mechanistic

## Abstract

Tusamitamab ravtansine (SAR408701) is an antibody-drug conjugate (ADC), combining a humanized monoclonal antibody (IgG1) targeting carcinoembryonic antigen-related cell adhesion molecule 5 (CEACAM5) and a potent cytotoxic maytansinoid derivative, DM4, inhibiting microtubule assembly. SAR408701 is currently in clinical development for the treatment of advanced solid tumors expressing CEACAM5. It is administered intravenously as a conjugated antibody with an average Drug Antibody Ratio (DAR) of 3.8. During SAR408701 clinical development, four entities were measured in plasma: conjugated antibody (SAR408701), naked antibody (NAB), DM4 and its methylated metabolite (MeDM4), both being active. Average DAR and proportions of individual DAR species were also assessed in a subset of patients. An integrated and semi-mechanistic population pharmacokinetic model describing the time-course of all entities in plasma and DAR measurements has been developed. All DAR moieties were assumed to share the same drug disposition parameters, excepted for clearance which differed for DAR0 (i.e. NAB entity). The conversion of higher DAR to lower DAR resulted in a DAR-dependent ADC deconjugation and was represented as an irreversible first-order process. Each conjugated antibody was assumed to contribute to DM4 formation. All data were fitted simultaneously and the model developed was successful in describing the pharmacokinetic profile of each entity. Such a structural model could be translated to other ADCs and gives insight of mechanistic processes governing ADC disposition. This framework will further be expanded to evaluate covariates impact on SAR408701 pharmacokinetics and its derivatives, and thus can help identifying sources of pharmacokinetic variability and potential efficacy and safety pharmacokinetic drivers.

**Supplementary Information:**

The online version contains supplementary material available at 10.1007/s10928-021-09799-0.

## Introduction

Delivering potent cytotoxics to tumor cells using antibody-drug conjugates (ADCs) has been shown to be an effective strategy for cancer therapy, as demonstrated by approvals of brentuximab vedotin (approved in 2011 for CD30-positive lymphomas [1]), trastuzumab emtansine (approved for HER2-positive advanced breast cancer in 2012 [2]), gemtuzumab ozogamicin (reapproved in 2017, after market withdrawal, for CD33-positive acute myeloid leukemia [3]), inotuzumab ozogamicin (in 2017, for CD22-positive B-cell precursor acute lymphoblastic leukaemia [4]), polatuzumab vedotin (in 2019, for relapsed diffuse large B-cell lymphoma [5]) and belantamab mafodotin, the latest approved ADC in 2020 for multiple myeloma patients [6]. Key steps of clinical development of approved ADCs are summarized in Liu and Li’s review [7].

The high number of ADC candidates (approximately 80 under clinical development) and the nearly 600 ongoing clinical trials [8] attest the growing interest toward such therapeutics and the hopes of a safer and more efficient antitumoral treatment.

SAR408701 is a first in class ADC directed against carcinoembryonic antigen-related cell adhesion molecule 5 (CEACAM5). CEACAM5 belongs to the human carcinoembryonic antigen family involved in cell adhesion, differentiation, proliferation, and survival [9, 10]. Known as a cell-surface glycoprotein, CEACAM5 is highly expressed in several epithelial tumors, including colorectal cancer, lung, and gastric adenocarcinoma. This antigen displays a limited expression in normal tissues of epithelial origin and can be found solely at the luminal surface of columnar absorptive cells. In tumor tissues, due to loss of cancer cell polarity, antigen distribution is extended around the cell [9]. CEACAM5 is therefore considered as an attractive target for drug delivery into tumors.

SAR408701 immunoconjugate combines a humanized monoclonal antibody (IgG1) targeting CEACAM5 and DM4, a potent maytansine derivative. The payload is covalently bound to the antibody via an N-succinimidyl 4-(2-pyridyldithio) butyrate (SPDB) linker, stable in plasma and cleavable inside cells after lysosomal degradation. DM4 acts as a potent antimitotic agent that induces mitotic arrest by inhibiting microtubule assembly and kills tumor cells [11].

After binding to CEACAM5 antigens, SAR408701 is internalized into cancer cells via antigen-mediated endocytosis. Cleavage occurs intracellularly, allowing release of the cytotoxic payload within the tumor cell. SAR408701 is degraded to form the lysine-linked derivative (lysine-SPDB-DM4) that gets further reduced in DM4. The free maytansinoid thiol derivative DM4 is rapidly methylated by an endogenous S-methyl transferase to form S-methyl-DM4 (MeDM4). A subsequent NADPH-dependent oxidation in liver yields the formation of the sulfoxide and sulfone derivatives, that are excreted into the bile [12]. All three metabolites (Lysine-SPBD-DM4, DM4 and MeDM4) have potent cytotoxic activity through binding to tubulin and inhibiting microtubule polymerization [13, 14].

Due to physicochemical properties of the SPBD linker, metabolites produced after SAR408701 degradation are neutrally charged and undergo passive diffusion into neighboring cells [15]. This so-called bystander effect allows cells that are distant from vessels and cells that do not express CEACAM5 to be exposed to DM4 and MeDM4 cytotoxics and thus potentiates anti-tumoral drug activity [16, 17]. As both DM4 and MeDM4 were observed as circulating entities after administration of previous ADC of the same construct (SAR3419: a mAb-SPDB-DM4 ADC [18]) they were hence quantified following SAR408701 administration.

Based on promising preclinical data presented by Decary et al. [19], SAR408701 appeared to be a promising candidate for clinical development. It is therefore currently tested in patients with advanced solid tumors expressing CEACAM5. SAR408701 is administered intravenously as a conjugated antibody containing species with different payload densities. The number of cytotoxic molecules per antibody is defined as the drug-to-antibody ratio (DAR). DAR distribution is heterogeneous and ranges from 0 to 8, with an average DAR in the administered solution of 3.8. During TED13751 first in human (FIH) clinical study (ClinicalTrials.gov Identifier: NCT02187848), SAR408701 (i.e., conjugated antibody with at least one payload: DAR≥1), unconjugated DM4 and MeDM4 were measured in plasma. While total antibody (i.e., conjugated antibody and naked antibody: DAR≥0) is usually measured during ADCs development, an innovative approach [20] allowed specific quantification of naked antibody (NAB) in TED13751 study. Moreover, new assays have also recently been developed [21, 22] to measure individual DAR moieties. DAR was therefore characterized in a subset of TED13751 patients and expressed as average DAR and proportions of individual DAR species over time.

Our objective was to elaborate a model framework that investigates SAR408701 disposition mechanisms, to support its ongoing clinical development. This model aimed to integrate ADC prior knowledge by fitting simultaneously PK data of SAR408701, NAB, DM4 and MeDM4, including DAR measurements in cancer patients. Outcomes of such model were to characterize each entity PK properties, to derive each entity individual exposure parameters to determine the best efficacy and safety PK driver, to support CMC for batches specification and subsequently it will allow investigation of sources of PK variability for each entity.

## Methods

### Clinical study

Data from TED13751 study were included in the analysis [23]. In this study, SAR408701 was administered as a single agent by intravenous infusion every 2 weeks (Q2W, 1 cycle=2 weeks) or every 3 weeks (Q3W, 1 cycle=3 weeks) in adult patients with advanced solid tumors. This open-label, non-randomized trial was divided in several cohorts regrouped in the escalation and expansion phases. Patients cohorts and doses are summarized in Table 1.

**Table 1:** Number of patients in TED13751 study per cohort and dose level

	Cohorts	Dose levels
Escalation cohorts	Main escalation Q2W (n=31)	5 mg/m^2^ (n=2) 10 mg/m^2^ (n=4) 20 mg/m^2^ (n=1) 40 mg/m^2^ (n=3) 80 mg/m^2^ (n=3) 100 mg/m^2^ (n=6) 120 mg/m^2^ (n=9) 150 mg/m^2^ (n=3)
	Escalation Q2W with loading dose at cycle 1 followed by 100 mg/m^2^ Q2W (n=28)	120 mg/m^2^ (n=3) 135 mg/m^2^ (n=4) 150 mg/m^2^ (n=8) 170 mg/m^2^ (n=13)
	Escalation Q3W (n=15)	120 mg/m^2^ (n=9) 150 mg/m^2^ (n=3) 170 mg/m^2^ (n=6) 190 mg/m^2^ (n=3)
Expansion cohorts	Colorectal cancer (n=46)	100 mg/m^2^ Q2W (n=180)
	Gastric carcinoma (n=16)	
	Non-squamous non-small cell lung cancer (NSCLC), high CEACAM5 expressors (CEACAM5 target expression ≥2+ in intensity in at least 50% of the tumor cell population, n=64)	
	Non-squamous NSCLC, low CEACAM5 expressors (CEACAM5 target expression ≥2+ in intensity in between ≥1% and <50% of the tumor cell population, n=28)	
	Small cell lung cancer (SCLC, n=26)	

This study was approved by the Medical Ethics Committee and conducted in accordance with the International Conference on Harmonization guidelines for Good Clinical Practice. All patients provided written informed consent.

### Bioanalytical methods

Blood samples for PK assessment were collected at specified time-points of each cycle, with full PK profile in most of patients at cycle 1 and cycle 4. Several analytes measured by different types of assays were used for population PK analysis.

#### SAR408701 concentrations

SAR408701 ADC concentrations were measured in plasma samples using a validated immunoassay performed on the Gyrolab xP platform. The assay quantified conjugated antibody with at least one DM4 payload covalently bound, up to a lower limit of quantification (LLOQ) of 0.500 µg/mL.

#### NAB concentrations

NAB concentrations were quantified using an innovative approach, previously developed for another ADC of the same construct [20]. This assay first required a pre-treatment, where SAR408701 was removed from the incurred samples by an immune and magnetic separation process that involved biotinylated anti-DM4 monoclonal antibodies immobilized on streptavidin beads. This first purification step was followed by a competitive immuno-enzymatic assay with a LLOQ of 1 µg/mL. To avoid bioanalytical interference of SAR408701 on NAB quantification, samples were diluted to reach SAR408701 concentrations below 15 µg/mL. Consequently, NAB LLOQ in TED13751 study varied from 1 to 9.60 µg/mL depending on SAR408701 concentration in each sample.

#### DM4 and MeDM4 concentrations

Unconjugated DM4 and MeDM4 concentrations were determined in acidified plasma samples, using a validated Liquid-Chromatography assay coupled with tandem Mass Spectrometry (LC-MS/MS). The LLOQ was 0.2 ng/mL for both analytes.

#### DAR quantification in plasma

Quantification of DAR in plasma samples was evaluated by Liquid Chromatography coupled with High Resolution Mass Spectrometry (LC-HRMS) in a subset of patients [21, 22, 24]. In this assay the relative intensities of each DAR moiety were quantified in plasma and proportions of individual DAR species were derived over time. Average DAR per sample was then calculated from each DAR distribution.

#### DAR quantification in administered drug solution

DAR was also assessed in 4 administered batches of TED13751 study by visible UV (UV/Vis) spectroscopy [25]. DAR value was determined in the administered solution, using measured absorbances of SAR408701 ADC and extinction coefficients of NAB and DM4.

### Population PK modeling

Population PK model included PK data of SAR408701, NAB, DM4, MeDM4, average DAR and proportions of individual DAR species over time. As all entities were fitted simultaneously, DM4, MeDM4 and NAB concentrations were converted to ADC molar equivalent (normalization by SAR408701 molecular mass, Table 2).

**Table 2:** SAR408701, NAB, DM4 and MeDM4 molecular weight

Entity	Molecular weight (g/mol)
SAR408701	150,000*
NAB	144,522
DM4	780
MeDM4	794

*SAR408701 molecular weight varies slightly as a function of DAR, but it was assumed that each drug conjugate contributed to a negligible amount of the global molecular weight. The 150,000 g/mol molecular weight used for SAR408701 was an average molecular weight determined for an ADC with three [linker-DM4] complexes attached to the antibody

PK parameters were assumed to follow a log-normal distribution and interindividual variability (IIV) was estimated on most of the parameters as follow:

$${P}_{i}={P}_{TV} \times \text{exp}\left({\eta }_{P,i}\right)$$

Where ${P}_{i}$ is the individual patient parameter, ${P}_{TV}$ the population parameter typical value and ${\eta }_{P,i}$ the individual random effect for this parameter, normally distributed with a variance ${{\omega }_{p}}^{2}$.

Residual variability was modelled with combined error model for SAR408701, proportional error model for DM4, MeDM4 and NAB, and additive error model for DAR as follow:

$${Obs}_{i}={f}_{i}+\left({a}_{i}+{b}_{i}\times {f}_{i}\right).{\sigma }_{i}$$

Where ${Obs}_{i}$ is the individual observation for a given entity, ${f}_{i}$ the predicted concentration for this entity, ${\sigma }_{i}$ a random effect with normal distribution, zero mean and unity standard deviation, ${a}_{i}$ the additive error term and ${b}_{i}$ the proportional error term.

Initially, combined error model was used for all analytes, but preliminary investigation indicated that the error models mentioned above provided the same fit as combined error models.

Parameters were estimated using the parametric nonlinear mixed-effect modelling software Monolix (2020R1) with stochastic approximation expectation-maximization algorithm (SAEM) combined to Markov Chain Monte Carlo procedure. Relative Standard Errors (RSE) were calculated via estimation of the Fisher Information Matrix and log-likelihood calculation was estimated using importance sampling method. Pre and post data processing were performed with R software (V 3.6.1).

### Model selection and evaluation

The best model was determined by Objective Function value, Bayesian Information Criteria (OF and BIC respectively), physiological relevance of parameter estimates, precision of estimates (RSE), visual inspection of the fitted individual profiles and overall goodness-of-fit (GOF) plots. GOF plots included plots of observed concentrations versus model population and individual predicted concentrations, as well as population and individual weighted residuals (PWRES and iWRES) versus time and versus model predicted concentrations.

The final model was qualified by inspection of prediction-corrected visual predictive check (pc-VPC) [26] and normal prediction distribution error (NPDE) [27] plots. NPDE and pc-VPC were performed with Monte-Carlo simulations. Five hundred datasets, identical in structure to the original dataset, were simulated. For pc-VPC, in each bin the observed and simulated data were normalized based on the typical population prediction for the median time in the bin to remove the variability coming from binning across independent variables. Final pc-VPC were presented at cycle 1 and at cycle 4 (rich data collected). Empirical percentiles of observed data were compared to theoretical percentiles (at a level of 90%). For a true model, empirical percentiles should remain within the corresponding prediction intervals.

### Model development

**Fig. 1:**
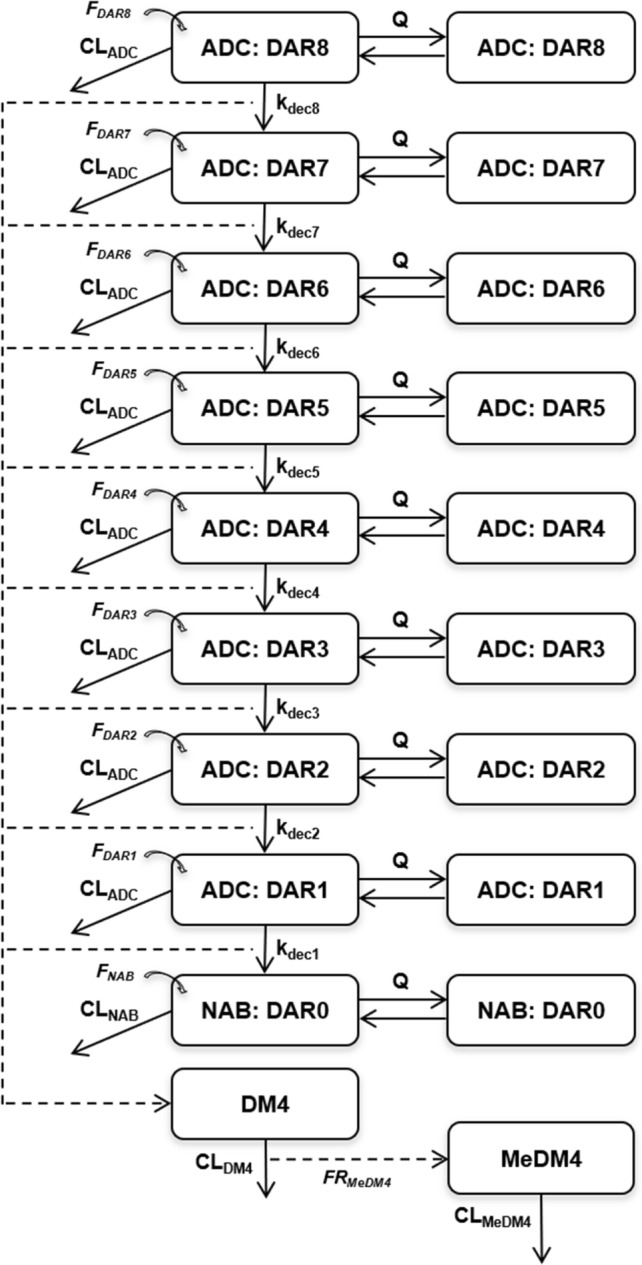
Semi-mechanistic pharmacokinetic model of tusamitamab ravtansine, a DM4 conjugated ADC

A semi-mechanistic integrated model characterizing the kinetics of all entities (SAR408701, DM4, MeDM4, NAB) in plasma, average DAR decrease over time and proportions of individual DAR species was developed and is presented Fig. 1. According to the parsimony rule, the model building was kept as simple as possible to ensure parameter identifiability and avoid overparameterization while reflecting mechanistic behaviour for each entity. Details of model ordinary differential equations (ODE) can be found in Online Resource 1 (initial condition for each ODE was set to zero).

The model can be presented in several building blocks:

#### ADC model

To characterize the PK of SAR408701, species from DAR1 (i.e. ADC with one DM4 molecule) to DAR8 (i.e. ADC with eight DM4 molecules) were explicitly represented. Each DAR species was described by a two-compartment PK model and assumed to share with other DAR species the same distribution (same value of central volume: V_c_, peripheral volume: V_p_ and intercompartmental clearance: Q) and the same proteolytic elimination (through central clearance parameter: CL_ADC_). CL_ADC_ was thus assumed to be DAR independent (a DAR dependent clearance model was tested but did not improve model prediction criteria).

Conversion of higher DAR to lower DAR species was modelled as an irreversible first-order process in central compartment. First-order rates (defined by k_dec,i_ parameters) were estimated for each conversion from DAR_n_ to DAR_n−1_. Due to very low levels of DAR7 and DAR8 proportions, k_dec7_ and k_dec8_ parameters could not be estimated accurately and were assumed to be equal to k_dec6_. Measurements of DAR8 relative proportion were excluded from the analysis as the observed data were close to zero in most patients and induced model instability. All k_dec,i_ parameters were assumed to have the same IIV. Attempts to have different IIV parameters for each k_dec,i_ did not improved the fit but resulted in poor precision of IIV estimates.

SAR408701 ADC concentration measured in plasma, was defined as follows:

$${\text{C}}_{\text{A}\text{D}\text{C}}={\text{C}}_{\text{D}\text{A}\text{R}1}+{\text{C}}_{\text{D}\text{A}\text{R}2}+{\text{C}}_{\text{D}\text{A}\text{R}3}+{\text{C}}_{\text{D}\text{A}\text{R}4}+{\text{C}}_{\text{D}\text{A}\text{R}5}+{\text{C}}_{\text{D}\text{A}\text{R}6}+{\text{C}}_{\text{D}\text{A}\text{R}7}+{\text{C}}_{\text{D}\text{A}\text{R}8}$$

Where ${C}_{ADC}$ is the estimated SAR408701 plasma concentration and ${C}_{DARi}$ the individual ADC DAR_i_ estimated plasma concentration.

Models with peripheral DM4 deconjugation and combined central and peripheral DM4 deconjugation were tested but did not improve model prediction criteria.

#### NAB model

NAB was represented as the DAR0 species and shared with conjugated antibody the same distribution parameters (V_c_, V_p_ and Q) but not the same clearance. A specific CL_NAB_ parameter was estimated. SAR408701 and NAB elimination were linear as no evidence of target-mediated drug disposition at the tested doses was found. Non-linear elimination and combined linear/non-linear elimination were nevertheless evaluated for each analyte but didn’t improved data fitting, thus confirming linear PK of conjugated antibody and NAB.

#### Administered DAR fraction

SAR408701 administered dose was fractioned between all DAR compartments. Initially, DAR administered fractions (F_DARi_) and there corresponding IIV were fixed at values measured in dose material batches: median values for parameter estimates and coefficients of variation for IIV (Table 3). As diagnostics plots revealed that NAB fit seemed biased, F_NAB_ was allowed to be estimated, but the total fraction was kept to 100% by proportional decrease of all other fractions (details can be found in Online Resource 1).

**Table 3:** Model input: Proportion of each DAR in the administered dose

DAR	Fraction of each DAR in 4 (out of 7) administered batches of TED13751 study [min-max]	Coefficient of variation (CV)
F_NAB_	[0.00–0.04]	91.3%
F_DAR1_	[0.08–0.10]	9.1%
F_DAR2_	[0.17–0.21]	9.1%
F_DAR3_	[0.21–0.24]	6.6%
F_DAR4_	[0.20–0.23]	5.9%
F_DAR5_	[0.13–0.16]	10.2%
F_DAR6_	[0.06–0.08]	13.2%
F_DAR7_	[0.02–0.04]	27.2%
F_DAR8_	[0.00–0.01]	66.7%

#### DM4 and MeDM4 models

DM4 disposition was described by a one compartment PK model, with first-order elimination. Each DAR≥1 deconjugation process was assumed to contribute to DM4 formation by releasing one DM4 molecule.

Models with CL_ADC_ contributing to DM4 formation were tested but did not improve model prediction criteria.

MeDM4 was modelled sequentially with a one compartment model and first order formation process directly from DM4 elimination.

DM4 and MeDM4 kinetics were characterized by the following ordinary differential equations:

$$\frac{{\text{d}\text{A}}_{\text{D}\text{M}4}}{\text{d}\text{t}}={[\text{k}}_{\text{d}\text{e}\text{c}1}.{\text{A}}_{\text{D}\text{A}\text{R}1}+{\text{k}}_{\text{d}\text{e}\text{c}2}.{\text{A}}_{\text{D}\text{A}\text{R}2}+{\text{k}}_{\text{d}\text{e}\text{c}3}.{\text{A}}_{\text{D}\text{A}\text{R}3}+{\text{k}}_{\text{d}\text{e}\text{c}4}.{\text{A}}_{\text{D}\text{A}\text{R}4}+{\text{k}}_{\text{d}\text{e}\text{c}5}.{\text{A}}_{\text{D}\text{A}\text{R}5}+{\text{k}}_{\text{d}\text{e}\text{c}6}.({\text{A}}_{\text{D}\text{A}\text{R}6}+{\text{A}}_{\text{D}\text{A}\text{R}7}+{\text{A}}_{\text{D}\text{A}\text{R}8})]- \frac{{\text{C}\text{L}}_{\text{D}\text{M}4}}{{\text{V}}_{\text{D}\text{M}4}}.{\text{A}}_{\text{D}\text{M}4}$$

$$\frac{{\text{d}\text{A}}_{\text{M}\text{e}\text{D}\text{M}4}}{\text{d}\text{t}}= {\text{F}\text{R}}_{\text{M}\text{e}\text{D}\text{M}4}.\frac{{\text{C}\text{L}}_{\text{D}\text{M}4}}{{\text{V}}_{\text{D}\text{M}4}}.{\text{A}}_{\text{D}\text{M}4} - \frac{{\text{C}\text{L}}_{\text{M}\text{e}\text{D}\text{M}4}}{{\text{V}}_{\text{M}\text{e}\text{D}\text{M}4}} . {\text{A}}_{\text{M}\text{e}\text{D}\text{M}4}$$

Where ${A}_{DM4}$ and ${A}_{MeDM4}$ represent the amount of DM4 and MeDM4 respectively, ${k}_{deci}$ the individual DAR deconjugated rate, ${CL}_{DM4}$ and ${CL}_{MeDM4}$ the apparent clearance of DM4 and MeDM4 respectively, ${V}_{DM4}$ and ${V}_{MeDM4}$ the distribution volume of DM4 and MeDM4 respectively (fixed to 1) and ${FR}_{MeDM4}$ the apparent relative fraction of DM4 elimination related to MeDM4 formation. Models with estimated ${V}_{DM4}$ and ${V}_{MeDM4}$were tested but did not improve the fit, likely due to formation-limited kinetics of DM4 and model inability to estimate ${FR}_{MeDM4}$ and V_MeDM4_ at the same time.

#### Average DAR prediction and individual DAR relative proportion

Derived average DAR, as a function of time, was defined as the ratio between total DM4 conjugated to SAR408701 over total antibody concentration (NAB and conjugated antibody), according to the following equations:

$${\text{C}}_{\text{T}\text{A}\text{B}}={\text{C}}_{\text{A}\text{D}\text{C}}+{\text{C}}_{\text{N}\text{A}\text{B}}$$

$${\text{D}\text{M}4}_{\text{c}\text{o}\text{n}\text{j}\text{u}\text{g}\text{a}\text{t}\text{e}\text{d}, \text{t}\text{o}\text{t}}= {\text{C}}_{\text{D}\text{A}\text{R}1}+{2\times \text{C}}_{\text{D}\text{A}\text{R}2}+{3\times \text{C}}_{\text{D}\text{A}\text{R}3}+{4\times \text{C}}_{\text{D}\text{A}\text{R}4}+{5\times \text{C}}_{\text{D}\text{A}\text{R}5}+{6\times \text{C}}_{\text{D}\text{A}\text{R}6}+{7\times \text{C}}_{\text{D}\text{A}\text{R}7}+{8\times \text{C}}_{\text{D}\text{A}\text{R}8}$$

$${\text{D}\text{A}\text{R}}_{\text{a}\text{v}\text{e}\text{r}\text{a}\text{g}\text{e}}= \frac{{\text{D}\text{M}4}_{\text{c}\text{o}\text{n}\text{j}\text{u}\text{g}\text{a}\text{t}\text{e}\text{d}, \text{t}\text{o}\text{t}}}{{\text{C}}_{\text{T}\text{A}\text{B}}}$$

Where ${C}_{TAB}$ represents the concentration of total antibody, ${DM4}_{conjugated, tot}$ the concentration of total conjugated DM4 and ${DAR}_{average}$ the average DAR predicted.

Proportions of individual DAR species were derived as followed:

$${NAB}_{proportion}=100\times \frac{{C}_{NAB}}{{C}_{TAB}}$$ ; $$DAR{1}_{proportion}=100\times \frac{{C}_{DAR1}}{{C}_{TAB}}$$ ; …

### Model simulations

The final semi-mechanistic model was used to simulate typical profiles of SAR408701, NAB, DM4 and MeDM4, in order to characterize each entity steady-state achievement and accumulation ratio based on Area Under the Curve between 2 cycles (AUC_TAU_). The simulated dose was 100 mg/m^2^ administered Q2W, up to cycle 50 (at which steady state is considered reached). Steady state was achieved when the difference between AUC_TAU_ at cycle_i_ and AUC_TAU_ at cycle_50_ was less than 5%. Accumulation ratio was defined for AUC_TAU_ and maximal concentration (C_max_) as the ratio between parameter at steady-state and parameter at cycle 1:

$${\text{R}\text{a}\text{c}\text{c}}_{\text{C}\text{max}}=\frac{\text{C}\text{max}, \text{s}\text{s}}{\text{C}\text{max}, \text{c}\text{y}\text{c}\text{l}\text{e}1}$$

$${\text{R}\text{a}\text{c}\text{c}}_{\text{AUC}\text{TAU}}=\frac{\text{AUC}\text{TAU}, \text{s}\text{s}}{\text{AUC}\text{TAU}, \text{c}\text{y}\text{c}\text{l}\text{e}1}$$

Terminal half-life was calculated for each entity after simulation of a typical single dose profile. Terminal half-life was as follow:

$$half-life= \frac{concentratio{n}_{t1}-concentratio{n}_{t2}}{t1-t2}$$

The final model was also used to simulate SAR408701 typical profiles from DAR0 to DAR8 to assess individual DAR kinetics and exposure over time.

## Results

### Data

Data from 254 patients of TED13751 FIH study were included in the analysis. PK data were collected over 58 cycles (i.e. 29 months), with a median value of 4 cycles per patient (i.e. 2 months). A total of 3746 SAR408701 plasma concentrations, 3740 DM4 plasma concentrations, 3734 MeDM4 plasma concentrations and 3734 NAB plasma concentrations were analysed. As values below the limits of quantification (BLQ) represented 1%, 36%, 13% and 31% of total SAR408701, DM4, MeDM4 and NAB concentrations respectively, they were included in the analysis and used as censored data. DAR was assessed in 13 patients out of the 254 patients enrolled in TED13751 study.

Spaghetti plots of SAR408701, DM4, MeDM4 and NAB observed concentrations over time at cycle 1 are presented in Online Resource 2a. Spaghetti plots of average DAR and proportions of individual DAR species over time after dose are presented in Online Resource 2b and 2c respectively. Individual DAR8 relative proportion was not quantifiable and is thus not represented in spaghetti plots and not fitted in the model.

### Semi-mechanistic integrated model

Parameters estimates of final semi-mechanistic model are presented in Table 4.

**Table 4:** Population pharmacokinetics parameter estimates from final semi-mechanistic model

Fixed effects		Standard deviation of the random effect, ${\omega }_{p}$ (RSE%)	Residual error (RSE%)
Parameter	Estimate (RSE%)		
F_DAR8_ (%)	0.9 (fixed)	^a^	${a}_{ADC}$ (µg/mL) = 1.03 (6)
F_DAR7_ (%)	2.8 (fixed)	0.272 (fixed)	${b}_{ADC}$ = 8.9% (3)
F_DAR6_ (%)	7.1 (fixed)	0.132 (fixed)	${b}_{DM4}$ = 33.5% (2)
F_DAR5_ (%)	14.2 (fixed)	0.102 (fixed)	${b}_{MeDM4}$ = 50.0% (2)
F_DAR4_ (%)	19.9 (fixed)	0.059 (fixed)	${b}_{NAB}$ = 26.0% (2)
F_DAR3_ (%)	21.8 (fixed)	0.066 (fixed)	${a}_{DA{R}_{average}}$ = 0.219 (6)
F_DAR2_ (%)	17.5 (fixed)	0.091 (fixed)	${a}_{DAR{7}_{proportion} }$(%) = 0.517 (9)
F_DAR1_ (%)	8.5 (fixed)	0.091 (fixed)	${a}_{DAR{6}_{proportion} }$(%) = 0.798 (7)
F_NAB_ (%)	7.1 (3)	0.418 (7)	${a}_{DAR{5}_{proportion} }$(%) = 1.47 (7)
CL_ADC_ (L/day)	0.392 (3)	0.469 (5)	${a}_{DAR{4}_{proportion} }$(%) = 2.53 (7)
V_c_ (L)	3.37 (2)	0.245 (5)	${a}_{DAR{3}_{proportion} }$(%) = 3.26 (8)
Q (L/day)	0.543 (5)	0.529 (8)	${a}_{DAR{2}_{proportion} }$(%) = 3.48 (8)
V_p_ (L)	2.54 (5)	0.605 (8)	${a}_{DAR{1}_{proportion} }$(%) = 4.58 (6)
k_dec8_ (/day)	0.938 (4)	0.202 (8)	${a}_{{NAB}_{proportion} }$(%) = 9.93 (6)
k_dec7_ (/day)			
k_dec6_ (/day)			
k_dec5_ (/day)	0.751 (3)		
k_dec4_ (/day)	0.525 (4)		
k_dec3_ (/day)	0.340 (4)		
k_dec2_ (/day)	0.181 (3)		
k_dec1_ (/day)	0.0565 (2)		
CL_NAB_ (L/day)	0.408 (3)	0.345 (6)	
CL_DM4_ (L/day)	240 (3)	0.365 (6)	
CL_MeDM4_ (L/day)	0.256 (5)	0.654 (6)	
FR_MeDM4_	0.0107 (5)	0.723 (5)	

^a^IIV on F_DAR8_ was back calculated to ensure equilibrium among administered DAR fractions

SAR408701 and NAB concentrations were well described by a combination of two-compartment models (one for each DAR). Distribution volumes were low (as expected for macromolecules) and close to physiological blood volume, with V_c_ estimated at 3.37 L and V_p_ at 2.54 L. DM4 and MeDM4 PK were well described by 1 compartment model and linear elimination. Apparent DM4 clearance was estimated at 240 L/day with 36.5% IIV and apparent MeDM4 clearance was estimated at 0.256 L/day with 65.4% IIV. F_NAB_ was estimated at 7.1%, with 41.8% IIV.

Deconjugation rate values were DAR dependent and increased from 0.0565 /day for k_dec1_ to 0.938 /day for k_dec6_, with higher DAR experiencing higher k_dec_ values. IIV was estimated at 20.2% for all k_deci_.

While they were allowed to be different, population estimates of SAR408701 and NAB proteolytic clearance (i.e. central clearance) were almost equal: SAR408701 proteolytic clearance was estimated at 0.392 L/d and NAB clearance at 0.408 L/day. Combining central deconjugation and proteolytic clearance, SAR408701 global clearance ranged from 0.582 L/d (for DAR1) to 3.55 L/d (for DAR≥6), with deconjugation clearance being the major elimination pathway for high DAR species (Table 5).

**Table 5:** ADC clearance pathways for each DAR

DAR	Proteolytic clearance (L/d)	Deconjugation clearance (L/d)	Global clearance (L/d)
DAR≥6	0.392	3.16	3.55
DAR5	0.392	2.53	2.92
DAR4	0.392	1.77	2.16
DAR3	0.392	1.15	1.54
DAR2	0.392	0.611	1.00
DAR1	0.392	0.190	0.582

### Model qualification

Visual inspection of individual fits showed good prediction of individual profiles for each entity. GOF plots and NPDE (Online Resource 3 and 4) confirmed good consistency between predicted and observed concentrations, with no apparent bias in residuals plots over time, suggesting that the semi-mechanistic model was successful in characterizing simultaneously PK of SAR408701, NAB, DM4, MeDM4 entities and DAR.

**Fig. 2:**
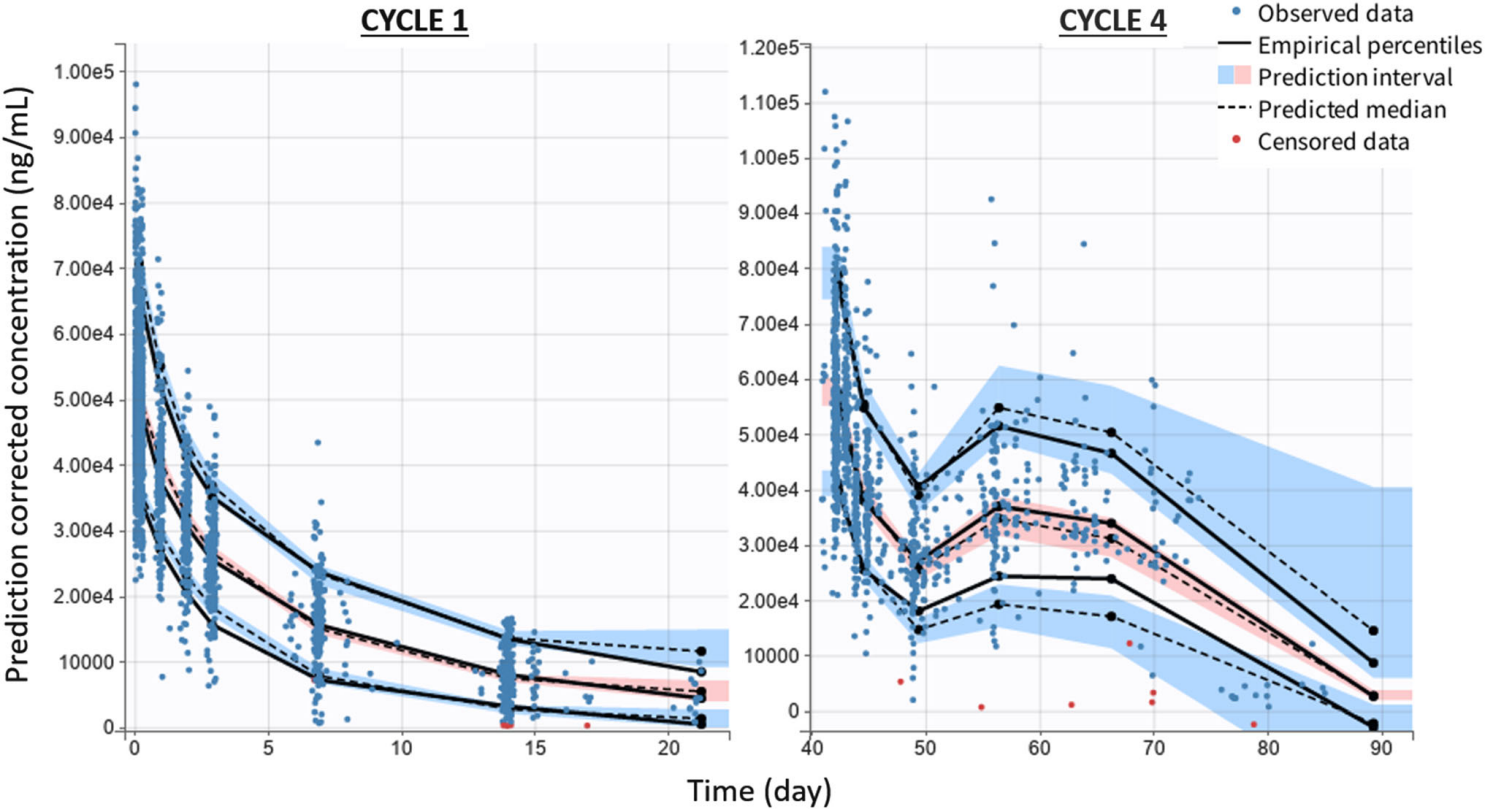
SAR408701 prediction corrected VPC at cycle 1 and cycle 4. The points represent the observed concentrations (in red the BLQ values, handled as censored data), the solid lines represent the median, 10th and 90th percentiles of the observed data and the blue and red areas represent the prediction intervals for each percentile (at a level of 90%). The shape of PK profiles may differ at cycle 4 because of dose delay that can be found across cycles for some patients

Prediction corrected visual predictive check showed that the 10th, 50th and 90th empirical percentiles of observed concentrations were in good agreement with the simulated confidence intervals for SAR408701 (Fig. 2). DM4, MeDM4 and NAB pc-VPC are presented in Online Resource 5.

All model parameters were estimated with good precision and magnitude of residual variability for each entity was low for ADC, NAB and DM4 (${b}_{ADC}$ = 8.9%, ${b}_{DM4}$ = 33.5%, ${b}_{NAB}$ = 26.0%) but higher for MeDM4 (${b}_{MeDM4}$ = 50.0%), indicating good overall fit and no model misspecification. The moderate estimated error on MeDM4 arises partly from the high variability observed on MeDM4 profiles (Online Resource 2a): observed C_max_ values range at cycle 1 from 0.00050 µM to 0.050 µM for some patients who experienced very high C_max_.

### Steady state and accumulation

**Fig. 3:**
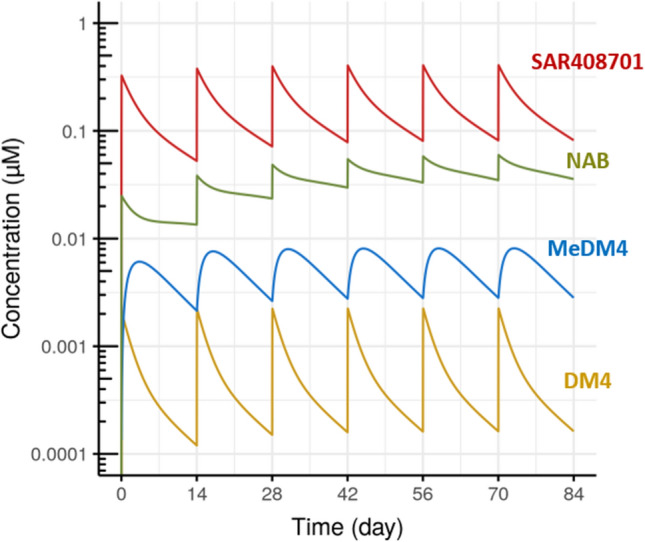
SAR408701, DM4, MeDM4 and NAB typical profile (after 100 mg/m^2^ Q2W dosing)

The final model was used to simulate typical profiles for each entity (Fig. 3). As expected, simulations show low levels of DM4, MeDM4 and NAB compared to conjugated antibody and rapid elimination of DM4 entity. Terminal half-life is estimated at 8.8 days for SAR408701 and 12.2 days for NAB. For DM4 and MeDM4, estimated terminal half-life values (8.7 days) are almost equal to that of ADC and thus reflects a formation limited kinetic process experienced by these two entities. Steady state is reached for Q2W administration at cycle 3 (i.e. day 42) for all entities except for NAB. NAB equilibrium is achieved at cycle 6 (i.e. day 84), with more accumulation than the other entities explained by the additional NAB input coming from successive DAR deconjugations. Racc for AUC_TAU_ ranges from 1.1 to 1.4 for SAR408701, DM4 and MeDM4 whereas it achieves 2.9 for NAB (Table 6). Typical exposure parameters at cycle 1 for all entities and comparisons with SAR408701 exposure (Table 7) confirm that DM4 and MeDM4 account for a very small proportion of SAR408701 exposure (less than 5%).

**Table 6:** Terminal half-life, steady-state and accumulation ratio for each entity (after 100 mg/m^2^ Q2W dosing)

Entity	Terminal half-life (days)	Steady-state	Racc for AUC_TAU_	Racc for C_max_
SAR408701	8.8	Cycle 3	1.4	1.3
DM4	8.7^a^	Cycle 3	1.1	1.1
MeDM4	8.7^a^	Cycle 3	1.3	1.3
NAB	12.2	Cycle 6	2.9	2.5

^a^Apparent terminal half-life: formation limited kinetic for DM4 and MeDM4

**Table 7:** Typical exposure parameters at cycle 1 for each entity (after 100 mg/m^2^ Q2W dosing)

Entity	Cycle 1 AUC_TAU, i_ (µM.day)	$\frac{\text{A}\text{U}{\text{C}}_{\text{T}\text{A}\text{U},\text{i}}}{\text{A}\text{U}{\text{C}}_{\text{T}\text{A}\text{U}, \text{S}\text{A}\text{R}408,701}}$	Cycle 1 C_max, i_ (µM)	$\frac{{\text{C}}_{\text{m}\text{a}\text{x}, \text{i}}}{{\text{C}}_{\text{m}\text{a}\text{x}, \text{S}\text{A}\text{R}408,701}}$
SAR408701	250	1	0.326	1
DM4	1.00	0.40%	0.00208	0.64%
MeDM4	8.69	3.5%	0.00610	1.9%
NAB	32.5	13%	0.0249	7.6%

### DAR individual typical profiles

**Fig. 4:**
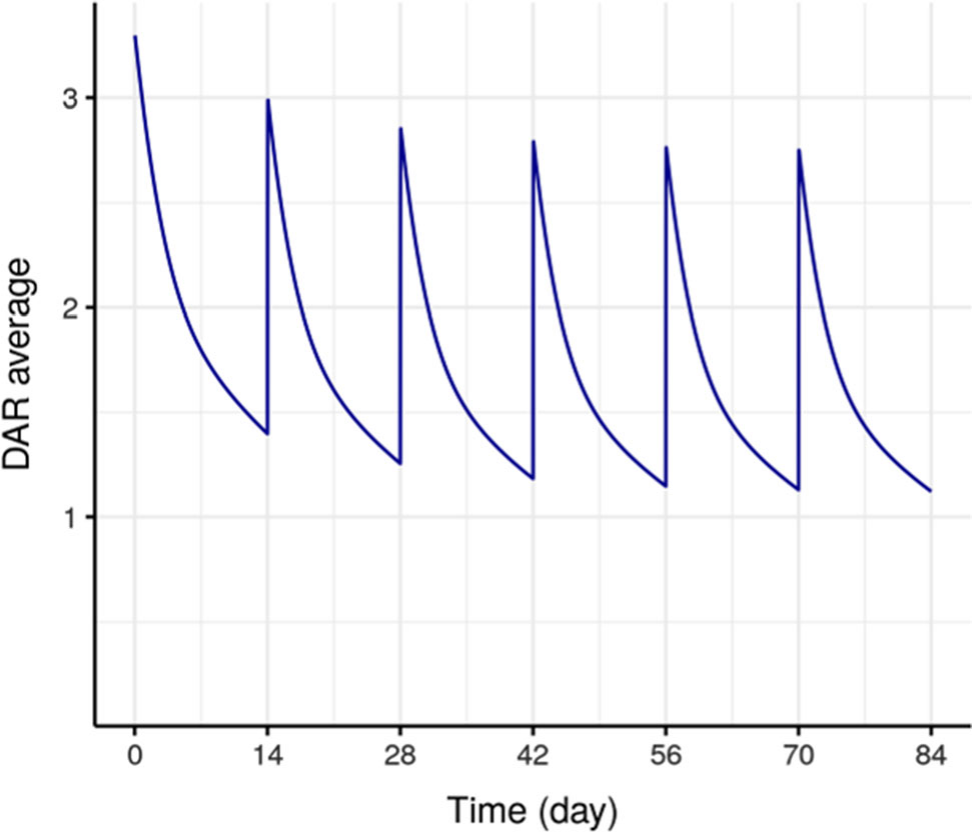
Typical average DAR profile (after 100 mg/m^2^ Q2W dosing)

**Fig. 5:**
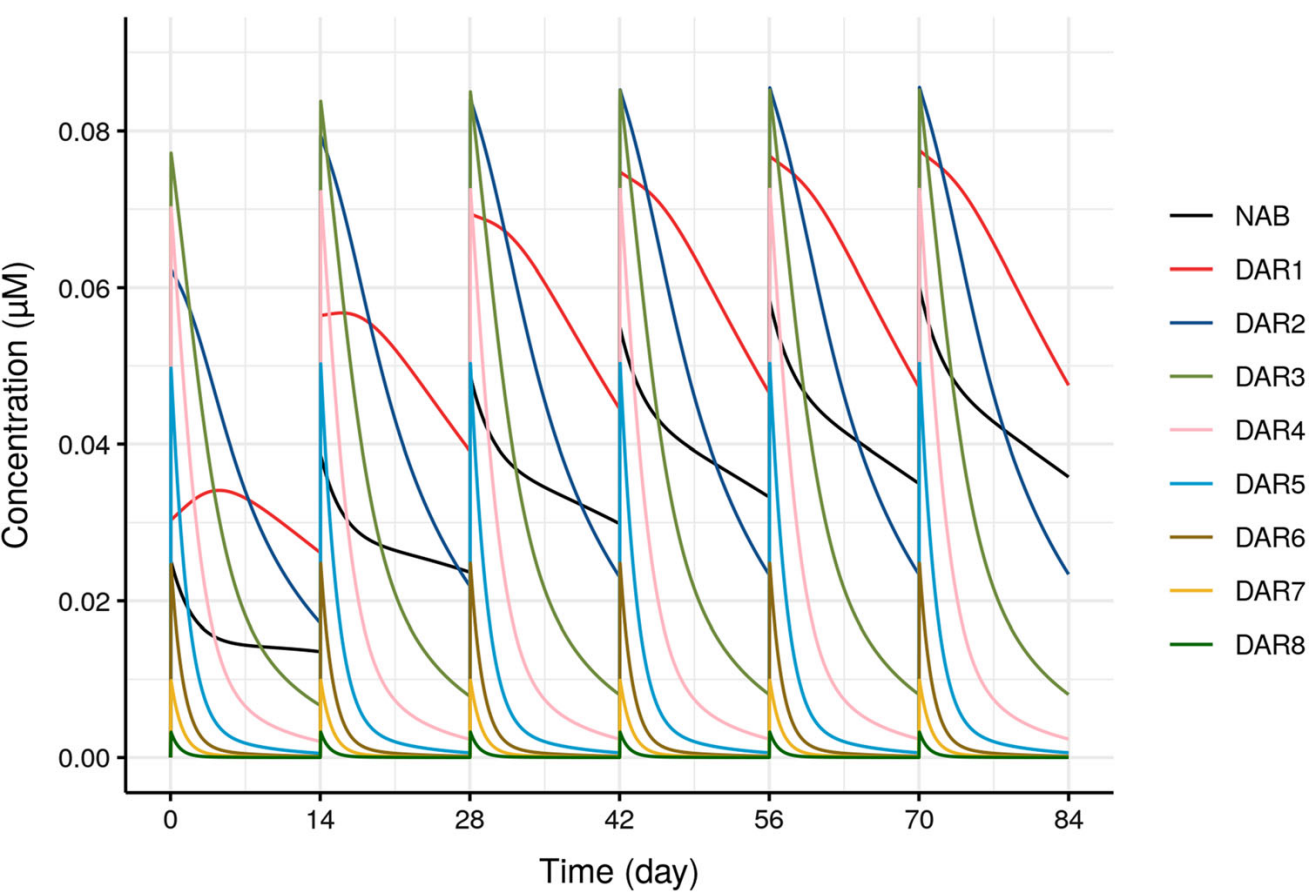
Typical individual DAR profiles (after 100 mg/m^2^ Q2W dosing)

Final model was also used to perform typical simulations of average DAR (Fig. 4) and individual DAR_i_ profiles (Fig. 5). After drug administration, low levels and fast decreases of high DAR species are observed, with no accumulation across cycles (Fig. 5). While for low DAR species, higher levels and slower decrease are observed with accumulation across cycles. Average DAR ranges across cycles from 1 to 3.3 (Fig. 4). The maximum average DAR reduces slightly from 3.3 to 2.8 after repeated doses of SAR408701 due to the accumulation of DAR0 and DAR1. Both graphs show that low DAR species (i.e. DAR1, DAR2, DAR3) are the major circulating species after drug administration.

## Discussion

PK modelling of ADC is challenging as the administered drug is a mixture of various DAR moieties that may have different kinetics and different contributions to payload release. Each DAR moiety is not commonly measured in clinical samples. PK information is often limited to conjugated antibody (ADC carrying at least 1 drug molecule) and total antibody (antibody irrespective of the drug load). An innovative analytical approach, developed by Pascual et al. [20], allows to selectively quantify naked antibody after ADC administration. Payloads and derived relevant metabolites are also generally measured, as they represent active entities.

A usual first approach to characterize the PK of an ADC and its derivatives is to analyse the PK of each entity separately. For instance, Hibma and Knight [28] studied the PK of gemtuzumab ozogamicin and elaborated separate models for total antibody and unconjugated payload. These models successfully described the PK of both entities. While being descriptive, this type of modelling approach may suffer from a lack of mechanistic basis which could limit the scope of further PK simulations.

SAR408701 is a DM4 conjugated antibody, bearing up to 8 DM4 molecules covalently bound to the antibody, with an average DAR of 3.8. Among the SAR408701 derivatives, DM4 and MeDM4 possess cytotoxic activity via tubulin binding and inhibition of tumor cell proliferation. These two entities are quantified in plasma at low levels: DM4 and MeDM4 exposures represent approximately 0.40% and 3.5% (on an AUC_TAU_ molar basis) of SAR408701 exposure, respectively. During the TED13751 study, conjugated antibody, NAB, DM4 and MeDM4 were measured in plasma in 254 patients. DAR was assessed in a subset of 13 patients and derived as average DAR and proportions of individual DAR species. The objectives of the current work were to explore the mechanisms governing SAR408701 disposition, characterize PK and quantify variability of all entities. This is the first published PK model of an anti-CEACAM5 ADC conjugated to DM4. All available data were integrated and fitted simultaneously in a semi-mechanistic model.

Similar modelling approaches have been considered previously to describe ADCs complex disposition. Semi-mechanistic models were explored and built with clinical or preclinical data, resulting in an enhanced comprehension of ADCs behaviour.

Gibiansky and Gibiansky [29] extended the target-mediated drug disposition (TMDD) model and its approximations to predict ADCs and released toxins PK. In their approach, ADC clearance and payload deconjugation rates could either be considered drug load dependent or independent. Their developed models were successful in describing clinical PK data simulated from trastuzumab emtansine (T-DM1) population PK models [30, 31].

Later on, Mittapalli et al. [32] adapted the Gibiansky and Gibiansky’s model to describe the PK of depatuxizumab mafodotin and its active metabolite. They developed a semi-mechanistic model integrating species from DAR0 to DAR8 and assumed that each deconjugation process produced 2 molecules of payload. They adequately characterized the PK profile of each entity and confirmed once again the value of such a mechanistic approach.

Semi-mechanistic models are also of interest during drug development to predict human PK from experimental preclinical data. Sukumaran et al. [33] presented an integrated model that included explicit representation of all DAR species, in which payload deconjugation and total antibody clearance were modelled as DAR dependent processes. Their model was built with rodents and cynomolgus monkeys data and adequately predicted human PK for an anti-STEAP-vs-MMAE ADC. Deng et al. [33] also described the human PK of a novel THIOMAB^TM^ antibody-antibiotic conjugate, with a cross species model built with mice, rats and monkeys data. The main question raised by these preclinical approaches remains the model translatability. While monkey allometric scaling appears to be a promising tool to predict human ADC PK in this mechanistic framework, translatability is still challenging, as multiple analytes and clearance pathways are involved and should be scaled.

All these models provide a surrogate mechanistic characterization of different DAR species, but individual DAR measurements are not usually included in these analyses. In fact, immunoassays commonly used measure total or conjugated antibody but fail to distinguish ADC components with different loads. Recent improvements in bioanalytical field discussed by Zhu et al. [22], allowed a deep characterization of ADC moieties by distinguishing the number of payloads attached to each ADC. Considering such DAR data in a mechanistic approach would enhance model confidence and allow a more accurate prediction of deconjugation processes.

Bender et al. [35] presented a preclinical mechanistic PK analysis of T-DM1 in which relative intensities of each DAR moiety in plasma were measured in monkeys by a novel affinity capture LC-MS assay [21, 24]. Calculated DAR intensities were converted to individual DAR plasma concentrations that were included and fitted in their model, to help elucidating the link between trastuzumab, T-DM1, and DAR measurements.

Based on these existing mechanistic frameworks, our model included representation of all DAR species after IV administration of a solution of SAR408701 to patients. On a physiological level, ADC deconjugation is known to depend on linker properties. SPBD linkers that attach DM4 payloads to SAR408701 antibody are designed to be stable in plasma and cleavable inside tumor cells. But despite their stability, deconjugation in plasma cannot be ruled out and is therefore described in our model. The deconjugation process was assumed to occur in central compartment only, as additional peripheral deconjugation didn’t improve model fit criteria. This assumption was also supported by Lu et al. [36] and Deng et al. [34] who observed that peripheral deconjugation in targeted tumor tissues could not be characterized separately and should be lumped with apparent central deconjugation. Besides, DM4 and MeDM4 released from deconjugation in tumor cells may diffuse through cellular membranes and reach the circulation. The model central deconjugation processes tried to account for all these physiological mechanisms governing ADC deconjugation.

In our analysis ADC deconjugation was found to be DAR dependent, with faster deconjugation rates estimated for high DAR species and slower deconjugation rates for low DAR species. This phenomenon of DAR dependent deconjugation is a process widely described in literature. Drug load is found to affect ADCs physical stability: Adem et al. [37] showed that for MMAE ADCs, high DAR were less stable than low DAR species. Moreover, in a mechanistic PK characterization of T-DM1, Bender et al. [35] showed that DM1 deconjugated fastest from the more highly loaded trastuzumab molecules. This phenomenon was also characterized with a Weibull distribution function [36] as a more flexible way to describe T-DM1 DAR dependent deconjugation.

Our model included a second elimination process, reflecting SAR408701 catabolism and proteolysis, via a central clearance parameter. Depending on ADC structure, clearance can be either DAR dependent or independent. Clearance of non-cleavable ADC is supposed to be less influenced by DAR: Sukumaran et al. [33] showed that ADCs with protease-cleavable linker experienced DAR dependent clearance, while Mittapalli et al. [32] modelled depatuxizumab mafodotin (designed with a noncleavable maleimido-caproyl linker) with DAR independent clearance. Since SAR408701 is composed of an optimized linker (stable in plasma but cleavable inside cells), its central clearance is expected to be DAR independent. While DAR dependent clearance was also tested in our model it didn’t improve the model fit, strengthening the reasonable assumption of a DAR independent elimination pathway for SAR408701.

This mechanistic model helped understanding the fate of each DAR moiety and allowed simulation of typical profiles for each DAR species. The simulations showed that high DAR resulted in low concentrations and underwent rapid elimination with no accumulation across cycles. Conversely, low DAR species experienced a slower plasma elimination and accumulated across cycles, thus generating higher concentrations of low DAR species at steady state. Another feature of such model is the simulation of various DAR inputs in the administered solution: evaluating the impact of different DAR distributions on individual DAR kinetics may support the specifications of the administered batches produced.

The estimated PK parameters of SAR408701 ADC were in good agreement with allometric prediction made by Bouillon-Pichault et al. [38]. Based on monkey and human PK data of two other ADCs of the same construct, they established allometric scaling for SAR408701 with monkey data and predicted human PK profile. This example confirms the interest toward translational strategy, as a valuable tool to design first in human clinical studies for ADCs.

All model parameters were well estimated and GOF plots showed good description of all entities PK profiles given the complexity of the analysis performed: not only the model fitted the 4 main analytes (SAR408701, NAB, DM4 and MeDM4), but also average DAR and proportions of individual DAR species. However, diagnostic plots of proportions of individual NAB species indicated a potential misfit of the model toward such data (Online Resource 3f). The administered NAB fraction was estimated at 7.1%, a little more than what was measured in some of the administered batches. Nevertheless, allowing the model to estimate this fraction improved prediction of NAB concentrations and overall model prediction criteria, without impacting estimated values of the other population parameters.

DM4 and MeDM4 were well described by sequential one compartment PK models, with linear elimination. DM4 apparent clearance was much higher than MeDM4 apparent clearance which resulted in much lower levels of DM4 concentrations. This is consistent with the known chemical properties of DM4: being a thiol bearing molecule, DM4 is very unstable and rapidly metabolized. DM4 and MeDM4 apparent terminal half-life values were equal to that of ADC, due to formation limited kinetics process. Indeed, so long half-life for small molecules would have been highly unusual. This formation limited kinetics process explains why distribution volumes couldn’t be estimated for these two entities that are not directly administered.

Based on this semi-mechanistic PK model, identification of potential covariates effect on conjugated antibody and payloads disposition is warranted. Another key application of this integrated model is that it allows prediction of individual PK profiles and drug exposure for each circulating entity (SAR408701, DM4 or MeDM4) that can either be tested as candidate for exposure vs. response analyses or integrated in a PK/PD modelling framework to determine efficacy and safety PK drivers.

## Conclusions

We developed a semi-mechanistic model that was able to describe the PK profiles of SAR408701 conjugated antibody and its derivatives (DM4, MeDM4 and NAB). Additionally, the model predicted the PK profiles of all the DAR species. This model, built with clinical data, integrated DAR measurements and specific NAB concentrations thanks to new bioanalytical methods. It aimed to improve understanding of the complex PK behaviour of DM4 conjugated ADCs. This model may be further used to explore sources of PK variabilities and define potential safety or efficacy PK drivers. As illustrated by many, this type of mechanistic framework is applicable to other ADCs formats, with different payloads or linker properties and can support any step of drug development.

## Supplementary Information

Below is the link to the electronic supplementary material.

Supplementary material 1 Monolix model (PDF 121.0 kb)

Supplementary material 2 Spaghetti plots (PDF 323.2 kb)

Supplementary material 3 GOF plots, observed data vs population and individual prediction (PDF 1129.8 kb)

Supplementary material 4 GOF plots, PWRES, iWRES and NPDE vs time and predicted concentrations (PDF 845.7 kb)

Supplementary material 5 pc-VPC at cycle 1 and at cycle 4 (PDF 460.7 kb)
