# Supplementary material for: Integrated multiple analytes and semi-mechanistic population pharmacokinetic model of tusamitamab ravtansine, a DM4 anti-CEACAM5 antibody-drug conjugate
Source: J Pharmacokinet Pharmacodyn. 2022 Feb 15;49(3):381–94. doi: 10.1007/s10928-021-09799-0 (PMC9098589; doi:10.1007/s10928-021-09799-0)
Supplement: Supplementary file 1 — Supplementary material 1 Monolix model (PDF 121.0 kb) [file 10928_2021_9799_MOESM1_ESM.pdf]

Integrated multiple analytes and semi-mechanistic population pharmacokinetic model of tusamitamab ravtansine, a DM4 anti-CEACAM5 antibody-drug conjugate

Journal of Pharmacokinetics and Pharmacodynamics

Clemence Pouzin, Leonid Gibiansky, Nathalie Fagniez, Michel Tod, Mustapha Chadjaa, Laurent Nguyen

Corresponding author: Clemence Pouzin, 1 Avenue Pierre Brossolette, 91380 Chilly-Mazarin, France. Email: Clemence.Pouzin@sanofi.fr

Supplemental material 1: Monolix model (.mlxtran)

```
[LONGITUDINAL]
input = {FR_DAR7, FR_DAR6, FR_DAR5, FR_DAR4, FR_DAR3, FR_DAR2, FR_DAR1, FR_NAB, CL_ADC, VC, Q,
VP, L6, L5, L4, L3, L2, k_dec1, CL_NAB, CL_DM4, CL_MeDM4, FR_MeDM4, V_DM4, V_MeDM4}


EQUATION:
;--------------------------
; DAR input fractions
;--------------------------
SUM = 1 + FR_DAR1 + FR_DAR2 + FR_DAR3 + FR_DAR4 + FR_DAR5 + FR_DAR6 + FR_DAR7 + FR_NAB
F_DAR8 = 1/SUM
F_DAR7 = FR_DAR7/SUM
F_DAR6 = FR_DAR6/SUM
F_DAR5 = FR_DAR5/SUM
F_DAR4 = FR_DAR4/SUM
F_DAR3 = FR_DAR3/SUM
F_DAR2 = FR_DAR2/SUM
F_DAR1 = FR_DAR1/SUM
F_NAB  = FR_NAB /SUM


PK:
compartment(cmt=1,   amount=A1,  concentration = C_ADC8c,    volume = VC)
compartment(cmt=2,   amount=A2,  concentration = C_ADC8p,    volume = VP)
compartment(cmt=3,   amount=A3,  concentration = C_ADC7c,    volume = VC)
compartment(cmt=4,   amount=A4,  concentration = C_ADC7p,    volume = VP)
compartment(cmt=5,   amount=A5,  concentration = C_ADC6c,    volume = VC)
compartment(cmt=6,   amount=A6,  concentration = C_ADC6p,    volume = VP)
compartment(cmt=7,   amount=A7,  concentration = C_ADC5c,    volume = VC)
compartment(cmt=8,   amount=A8,  concentration = C_ADC5p,    volume = VP)
compartment(cmt=9,   amount=A9,  concentration = C_ADC4c,    volume = VC)
compartment(cmt=10,  amount=A10, concentration = C_ADC4p,    volume = VP)
compartment(cmt=11,  amount=A11, concentration = C_ADC3c,    volume = VC)
compartment(cmt=12,  amount=A12, concentration = C_ADC3p,    volume = VP)
compartment(cmt=13,  amount=A13, concentration = C_ADC2c,    volume = VC)
compartment(cmt=14,  amount=A14, concentration = C_ADC2p,    volume = VP)
compartment(cmt=15,  amount=A15, concentration = C_ADC1c,    volume = VC)
compartment(cmt=16,  amount=A16, concentration = C_ADC1p,    volume = VP)
compartment(cmt=17,  amount=A17, concentration = C_NABc,     volume = VC)
compartment(cmt=18,  amount=A18, concentration = C_NABp,     volume = VP)
compartment(cmt=19,  amount=A19, concentration = C_DM4,      volume = V_DM4)
compartment(cmt=20,  amount=A20, concentration = C_MeDM4,    volume = V_MeDM4)
iv(cmt = 1, p = F_DAR8)
iv(cmt = 3, p = F_DAR7)
iv(cmt = 5, p = F_DAR6)
iv(cmt = 7, p = F_DAR5)
iv(cmt = 9, p = F_DAR4)
iv(cmt = 11,p = F_DAR3)
iv(cmt = 13,p = F_DAR2)
iv(cmt = 15,p = F_DAR1)
iv(cmt = 17,p = F_NAB)

EQUATION:
odeType=stiff

;--------------------------
; Constant definition
;--------------------------
k12   = Q/VC
k21   = Q/VP
k34   = Q/VC
k43   = Q/VP
k56   = Q/VC
k65   = Q/VP
k78   = Q/VC
k87   = Q/VP
k9_10  = Q/VC
k10_9  = Q/VP
k11_12 = Q/VC
k12_11 = Q/VP
k13_14 = Q/VC
k14_13 = Q/VP
k15_16 = Q/VC
k16_15 = Q/VP

k17_18 = Q/VC
k18_17 = Q/VP

k10 = CL_ADC/VC
k30 = CL_ADC/VC
k50 = CL_ADC/VC
k70 = CL_ADC/VC
k90 = CL_ADC/VC
k11_0 = CL_ADC/VC
k13_0 = CL_ADC/VC
k15_0 = CL_ADC/VC
k17_0 = CL_NAB/VC

k19_0 = CL_DM4/V_DM4
k20_0 = CL_MeDM4/V_MeDM4
```

```
;--------------------------
; Deconjugation
;--------------------------
k_dec8    = k_dec1 * L6
k_dec7    = k_dec1 * L6
k_dec6    = k_dec1 * L6
k_dec5    = k_dec1 * L5
k_dec4    = k_dec1 * L4
k_dec3    = k_dec1 * L3
k_dec2    = k_dec1 * L2

k13     = k_dec8
k35     = k_dec7
k57     = k_dec6
k79     = k_dec5
k9_11   = k_dec4
k11_13 = k_dec3
k13_15 = k_dec2
k15_17 = k_dec1

;-------------------------
; ODE and initial conditions
;-------------------------

A1_0    = 0
A2_0    = 0
A3_0    = 0
A4_0    = 0
A5_0    = 0
A6_0    = 0
A7_0    = 0
A8_0    = 0
A9_0    = 0
A01_0   = 0

A11_0   = 0
A12_0   = 0
A13_0   = 0
A14_0   = 0
A15_0   = 0
A16_0   = 0
A17_0   = 0
A18_0   = 0
A19_0   = 0
A20_0   = 0

ddt_A1  = -(k10+k12)*A1 - k13*A1 + k21*A2                    ;DAR8 CENTRAL
ddt_A2  = k12*A1-k21*A2                                       ;DAR8 PERIPHERAL

ddt_A3  = k13*A1 - (k30+k12)*A3 - k35*A3 + k21*A4             ;DAR7 CENTRAL
ddt_A4  = k12*A3-k21*A4                                       ;DAR7 PERIPHERAL

ddt_A5  = k35*A3 - (k50+k12)*A5 - k57*A5 + k21*A6             ;DAR6 CENTRAL
ddt_A6  = k12*A5-k21*A6                                       ;DAR6 PERIPHERAL

ddt_A7  = k57*A5 - (k70+k12)*A7 - k79*A7 + k21*A8             ;DAR5 CENTRAL
ddt_A8  = k12*A7-k21*A8                                       ;DAR5 PERIPHERAL

ddt_A9  = k79*A7 - (k90+k12)*A9 - k9_11*A9 + k21*A10          ;DAR4 CENTRAL
ddt_A10 = k12*A9-k21*A10                                      ;DAR4 PERIPHERAL

ddt_A11 = k9_11*A9 - (k11_0+k12)*A11 - k11_13*A11 + k21*A12   ;DAR3 CENTRAL
ddt_A12 = k12*A11-k21*A12                                     ;DAR3 PERIPHERAL

ddt_A13 = k11_13*A11 - (k13_0+k12)*A13 - k13_15*A13 + k21*A14  ;DAR2 CENTRAL
ddt_A14 = k12*A13-k21*A14                                     ;DAR2 PERIPHERAL

ddt_A15 = k13_15*A13 - (k15_0+k12)*A15 - k15_17*A15 + k21*A16  ;DAR1 CENTRAL
ddt_A16 = k12*A15-k21*A16                                     ;DAR1 PERIPHERAL

ddt_A17 = k15_17*A15 - (k17_0+k12)*A17 + k21*A18              ;DAR0 CENTRAL
ddt_A18 = k12*A17-k21*A18                                     ;DAR0 PERIPHERAL

input_1 = k_dec8*A1 + k_dec7*A3 + k_dec6*A5 + k_dec5*A7 + k_dec4*A9 + k_dec3*A11 + k_dec2*A13 +
k_dec1*A15
ddt_A19 = input_1  - k19_0*A19                                ;DM4 CENTRAL
ddt_A20 = FR_MeDM4 * k19_0*A19 - k20_0*A20                    ;MEDM4 CENTRAL


;--------------------------
; ENTITIES FIT
;--------------------------
C_ADC    = C_ADC8c + C_ADC7c + C_ADC6c + C_ADC5c + C_ADC4c + C_ADC3c + C_ADC2c + C_ADC1c
C_TAB    = C_ADC+C_NABc
DM4tot   = 8*C_ADC8c + 7*C_ADC7c + 6*C_ADC6c + 5*C_ADC5c + 4*C_ADC4c + 3*C_ADC3c + 2*C_ADC2c +
1*C_ADC1c
DAR      = DM4tot/C_TAB

prop_ADC8 = 100*C_ADC8c/C_TAB
prop_ADC7 = 100*C_ADC7c/C_TAB
prop_ADC6 = 100*C_ADC6c/C_TAB
prop_ADC5 = 100*C_ADC5c/C_TAB
prop_ADC4 = 100*C_ADC4c/C_TAB

prop_ADC3 = 100*C_ADC3c/C_TAB
prop_ADC2 = 100*C_ADC2c/C_TAB
prop_ADC1 = 100*C_ADC1c/C_TAB
prop_NAB  = 100*C_NABc /C_TAB


OUTPUT:
output = {C_ADC, C_DM4, C_MeDM4, C_NABc, DAR, prop_ADC8, prop_ADC7, prop_ADC6, prop_ADC5,
prop_ADC4, prop_ADC3, prop_ADC2, prop_ADC1, prop_NAB}
```
