# Supplementary material for: Integrated multiple analytes and semi-mechanistic population pharmacokinetic model of tusamitamab ravtansine, a DM4 anti-CEACAM5 antibody-drug conjugate
Source: J Pharmacokinet Pharmacodyn. 2022 Feb 15;49(3):381–94. doi: 10.1007/s10928-021-09799-0 (PMC9098589; doi:10.1007/s10928-021-09799-0)

Integrated multiple analytes and semi-mechanistic population pharmacokinetic model of tusamitamab ravtansine, a DM4 anti-CEACAM5 antibody-drug conjugate

Journal of Pharmacokinetics and Pharmacodynamics

Clemence Pouzin, Leonid Gibiansky, Nathalie Fagniez, Michel Tod, Mustapha Chadjaa, Laurent Nguyen

Corresponding author: Clemence Pouzin, 1 Avenue Pierre Brossolette, 91380 Chilly-Mazarin, France. Email: Clemence.Pouzin@sanofi.fr

Online Resource 2: Spaghetti plots

a. SAR408701, DM4, MeDM4 and NAB at cycle 1

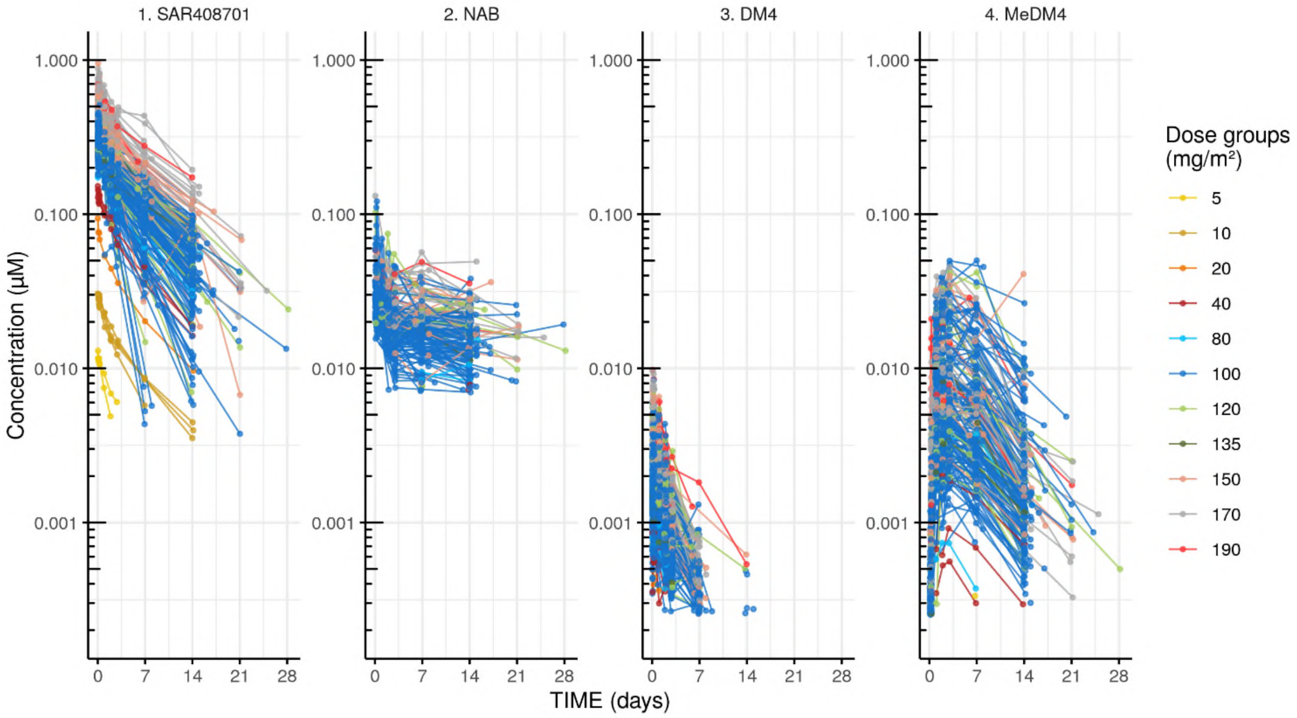

b. Average DAR data at cycle 1 and cycle 4.

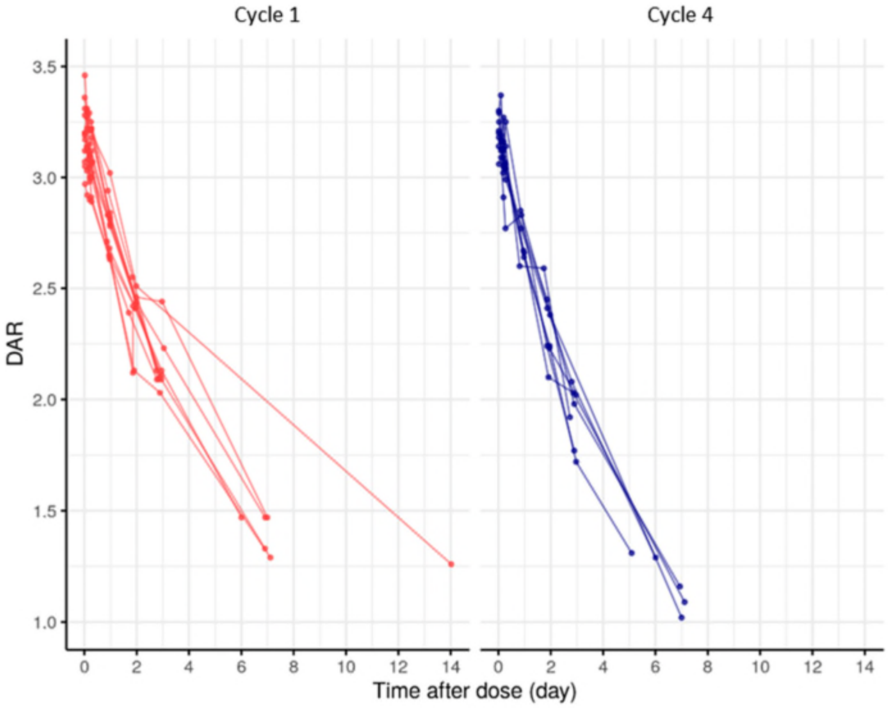

c. Proportions of individual DAR species at cycle 1 and cycle 4

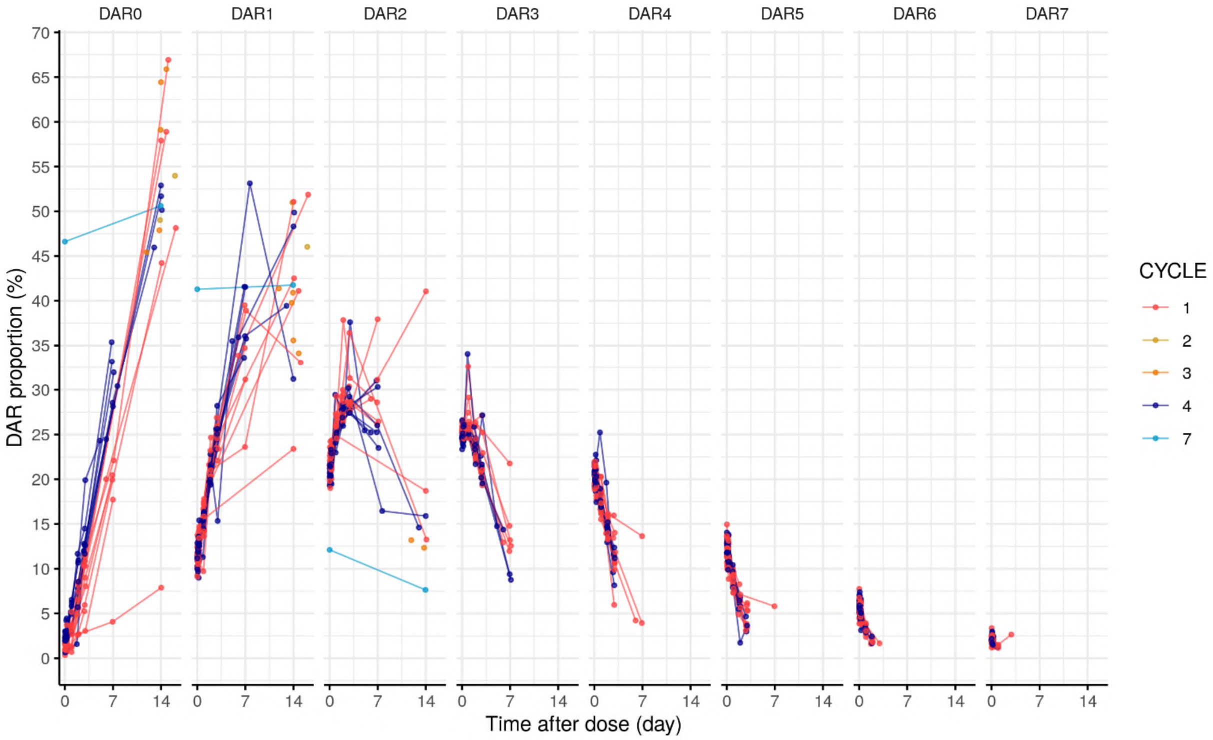

Supplement: Supplementary file 2 — Supplementary material 2 Spaghetti plots (PDF 323.2 kb) [file 10928_2021_9799_MOESM2_ESM.pdf]
