# Supplementary material for: Integrated multiple analytes and semi-mechanistic population pharmacokinetic model of tusamitamab ravtansine, a DM4 anti-CEACAM5 antibody-drug conjugate
Source: J Pharmacokinet Pharmacodyn. 2022 Feb 15;49(3):381–94. doi: 10.1007/s10928-021-09799-0 (PMC9098589; doi:10.1007/s10928-021-09799-0)

Integrated multiple analytes and semi-mechanistic population pharmacokinetic model of tusamitamab ravtansine, a DM4 anti-CEACAM5 antibody-drug conjugate

Journal of Pharmacokinetics and Pharmacodynamics

Clemence Pouzin, Leonid Gibiansky, Nathalie Fagniez, Michel Tod, Mustapha Chadjaa, Laurent Nguyen

Corresponding author: Clemence Pouzin, 1 Avenue Pierre Brossolette, 91380 Chilly-Mazarin, France. Email: Clemence.Pouzin@sanofi.fr

Online Resource 3: GOF plots, observed data vs population and individual prediction

a. SAR408701

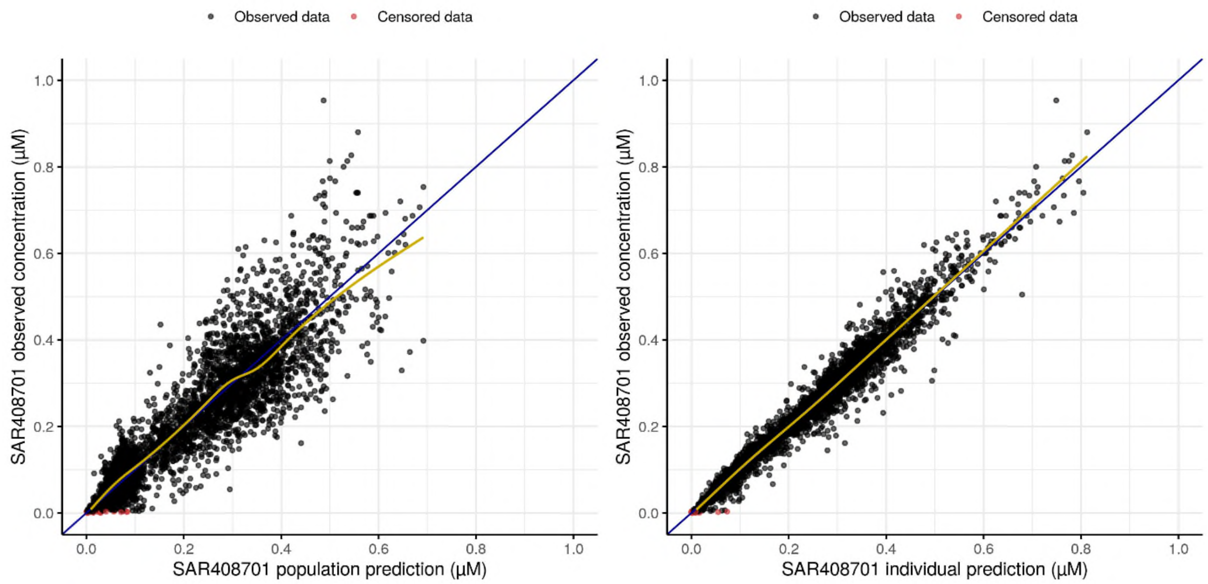

b. DM4

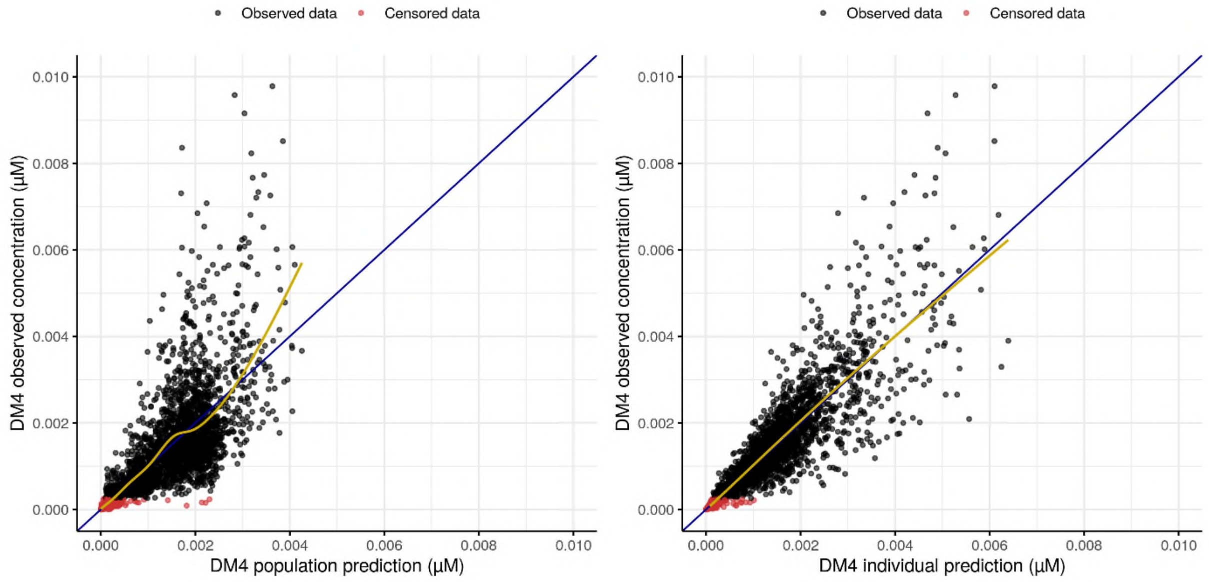

c. MeDM4

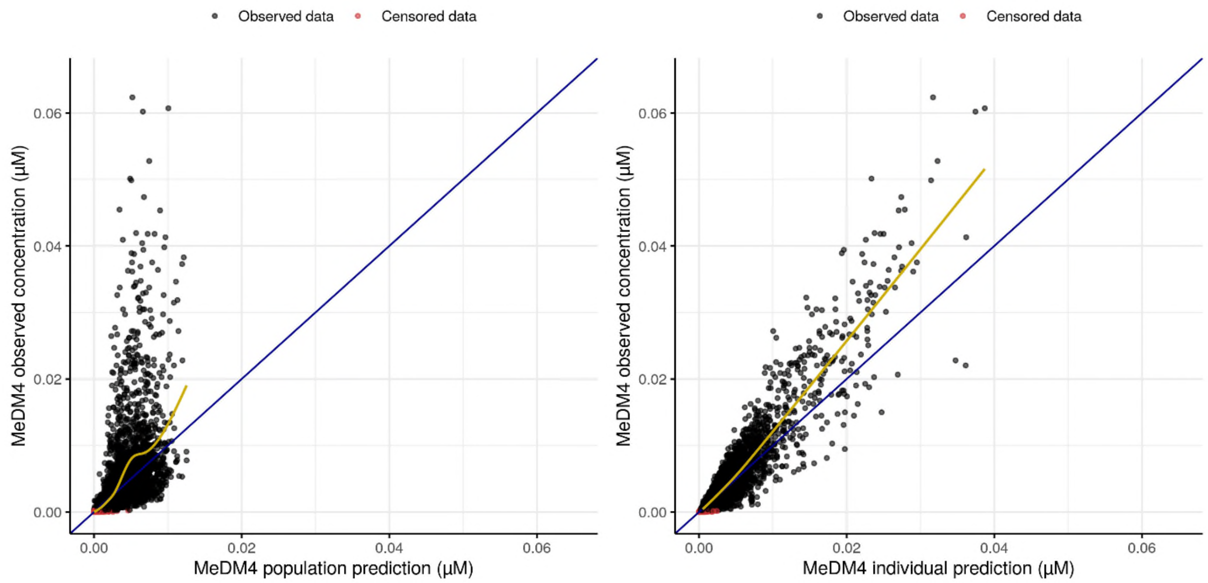
Observed data
Censored data
MeDM4 observed concentration (µM)
0.06
0.04
0.02
0.00
0.00
0.02
0.04
0.06
MeDM4 population prediction (µM)
Observed data
Censored data
MeDM4 observed concentration (µM)
MeDM4 individual prediction (µM)

d. NAB

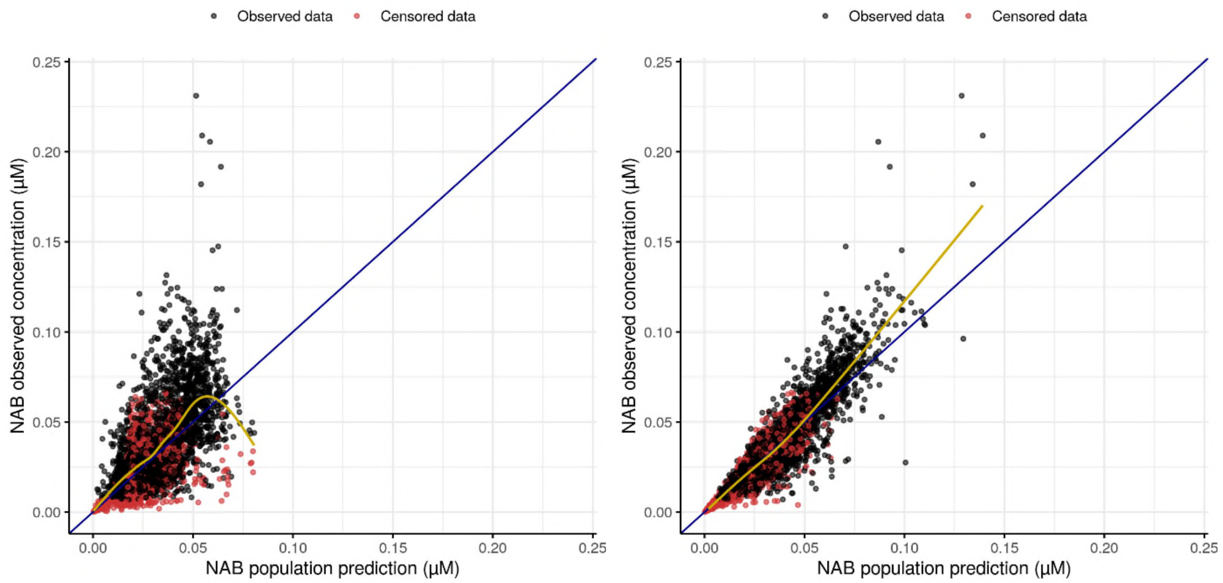
Observed data
Censored data
NAB observed concentration (µM)
0.25
0.20
0.15
0.10
0.05
0.00
0.00
0.05
0.10
0.15
0.20
0.25
NAB population prediction (µM)
Observed data
Censored data
NAB observed concentration (µM)
NAB population prediction (µM)

e. Average DAR

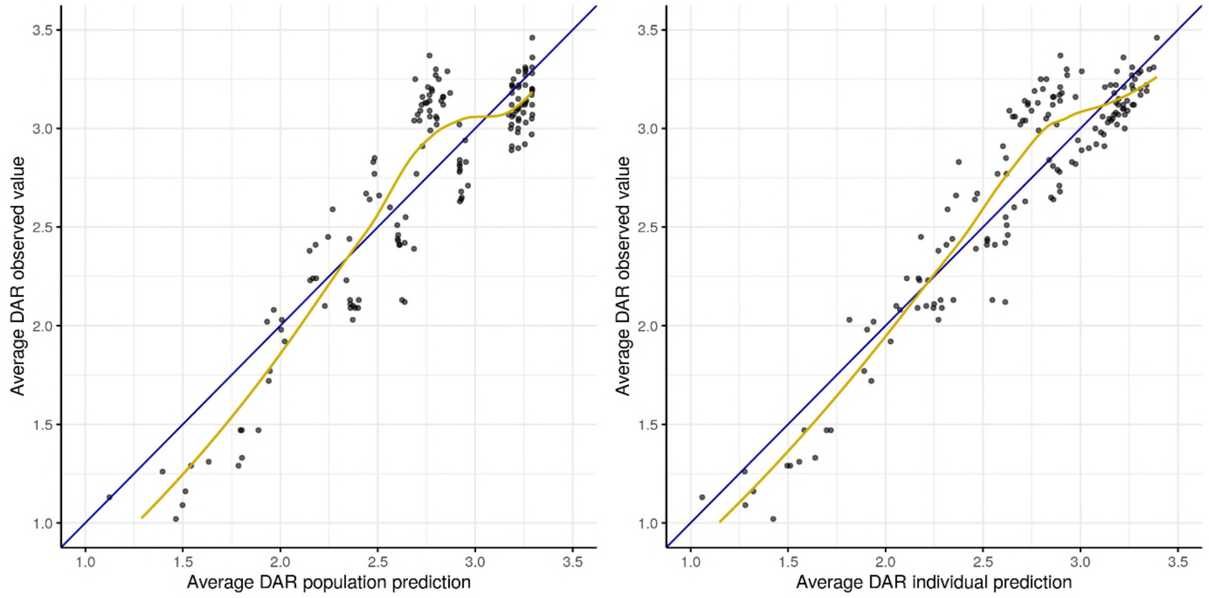

f. Proportions of individual DAR species: NAB (DAR0)

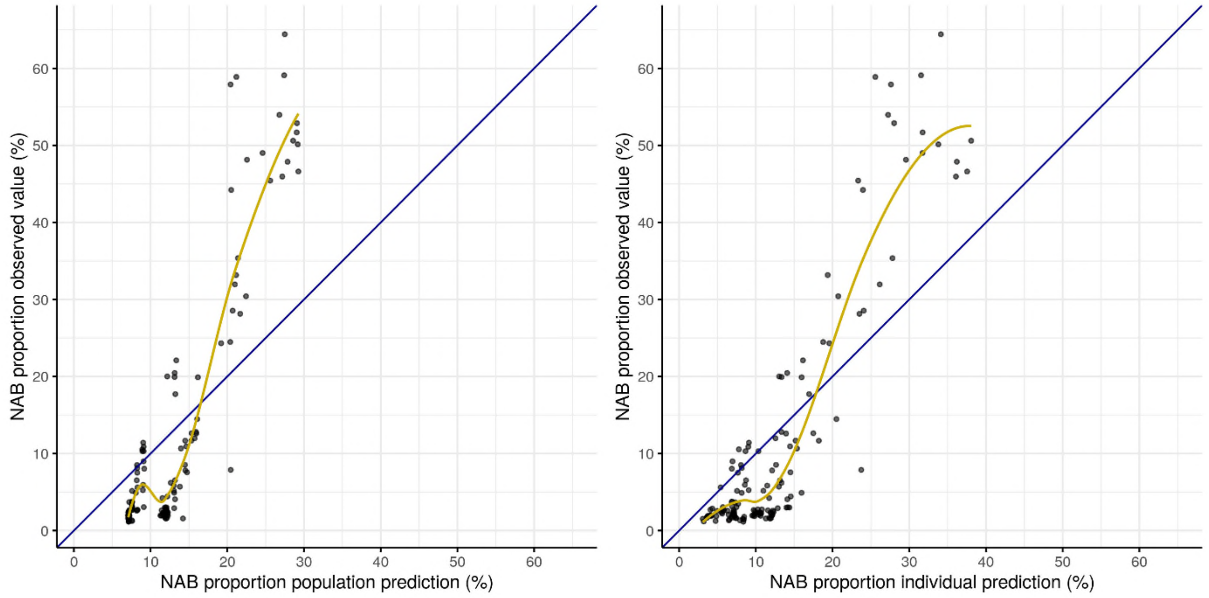

g. Proportions of individual DAR species: DAR1

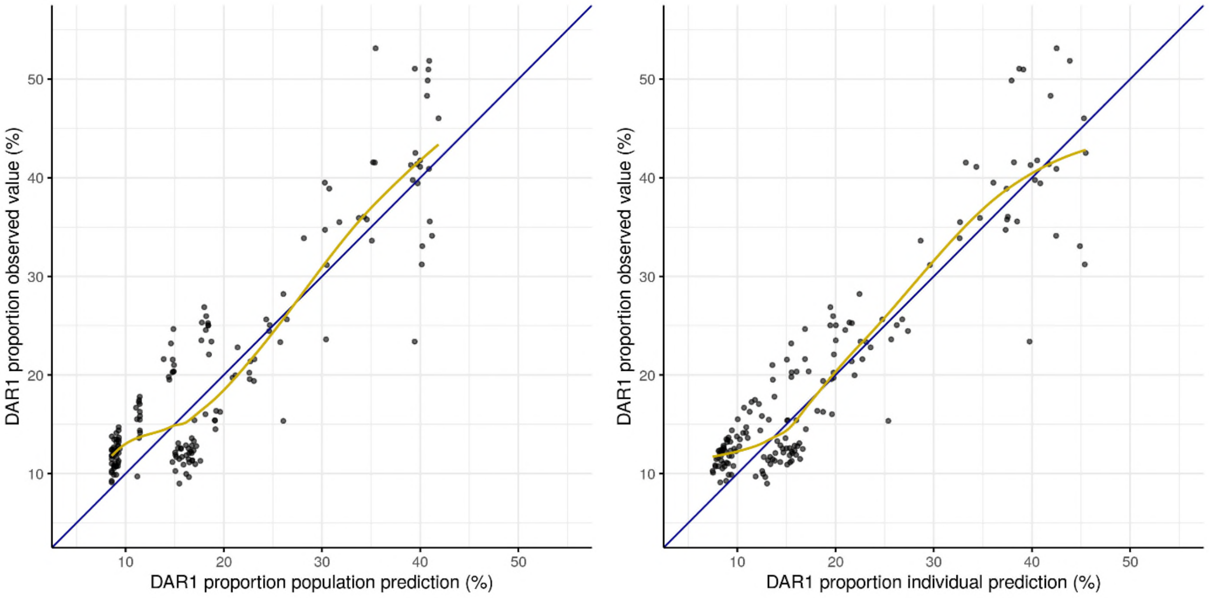

h. Proportions of individual DAR species: DAR2

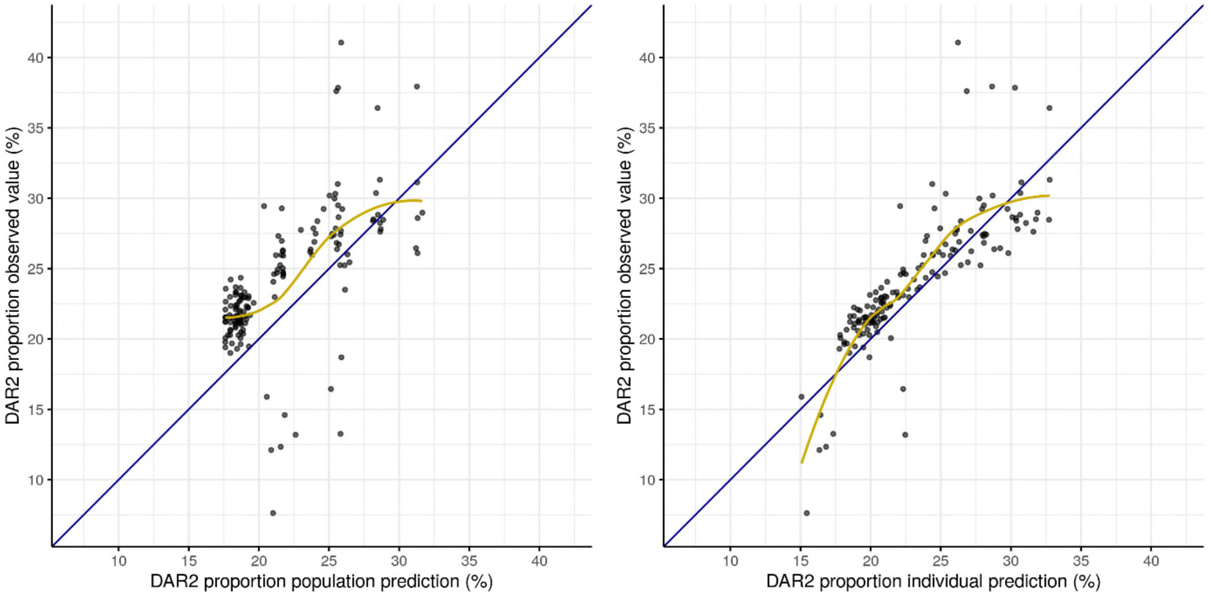

i. Proportions of individual DAR species: DAR3

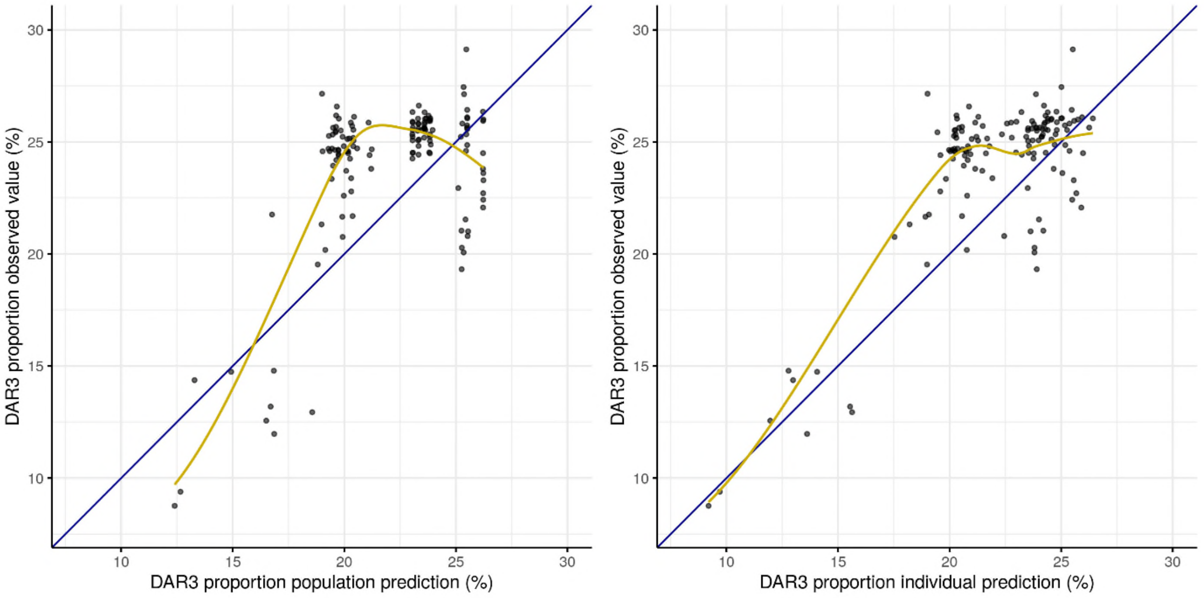

j. Proportions of individual DAR species: DAR4

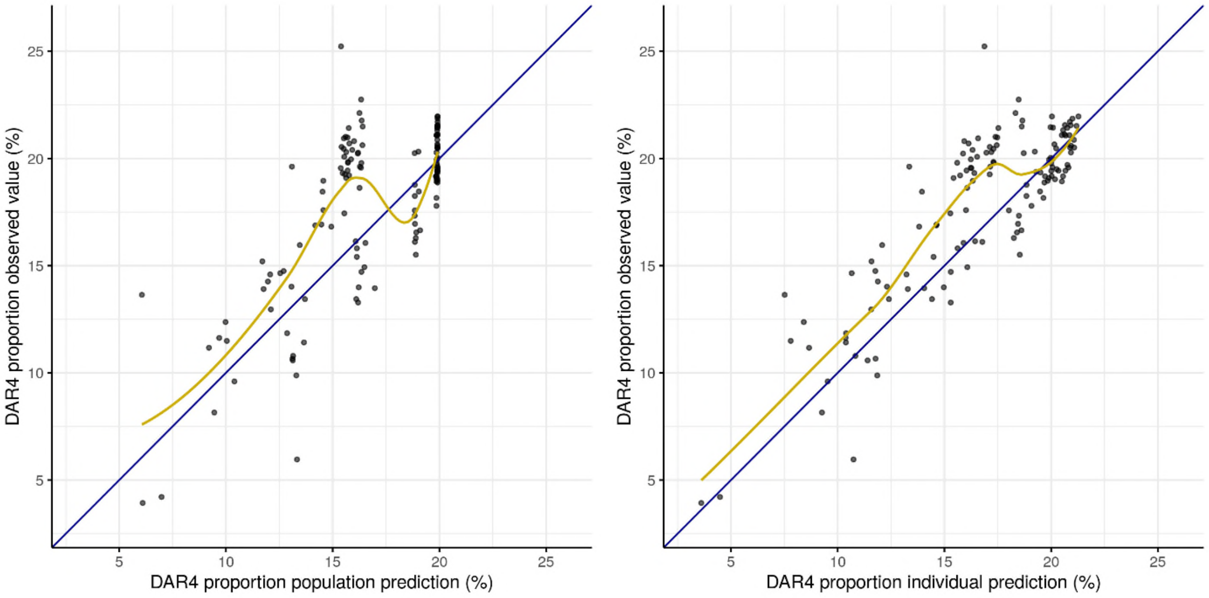

k. Proportions of individual DAR species: DAR5

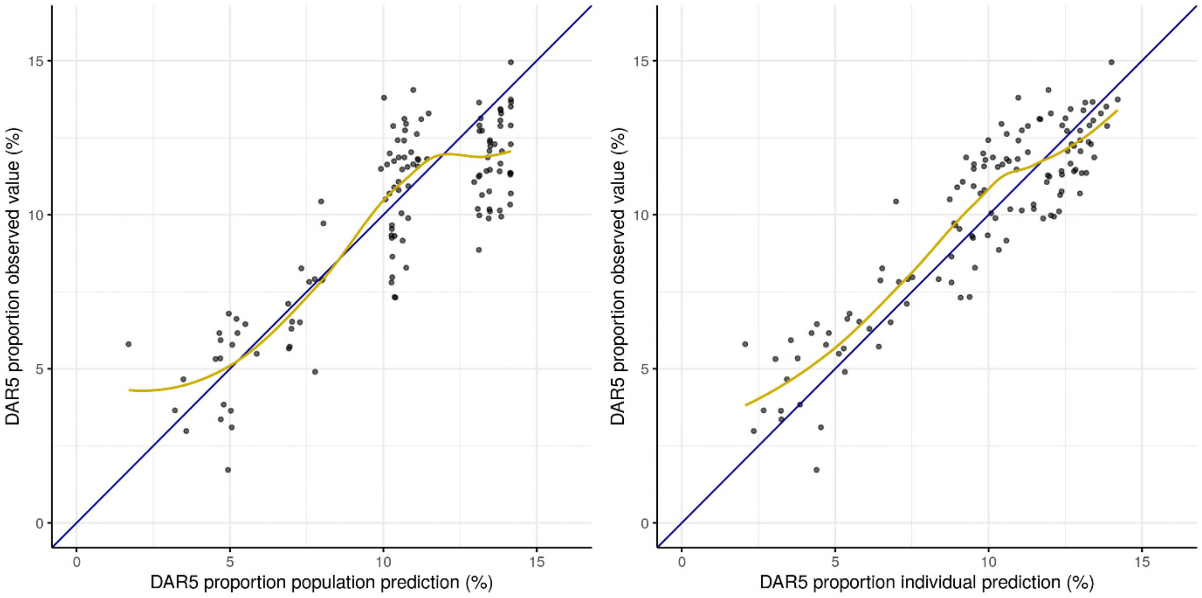

l. Proportions of individual DAR species: DAR6

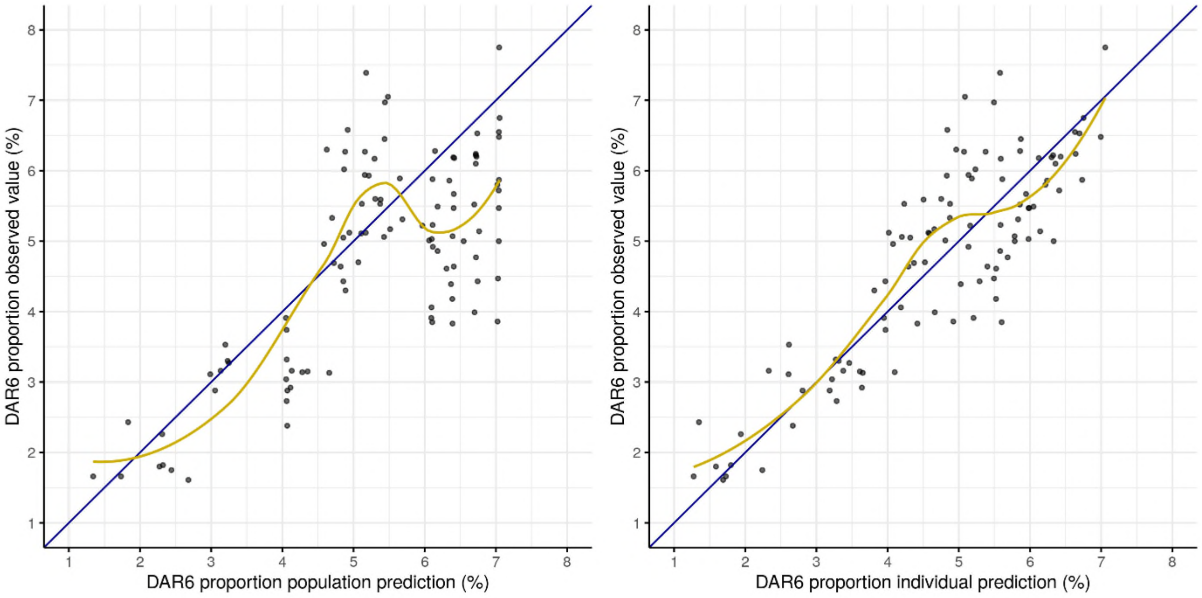

m. Proportions of individual DAR species: DAR7

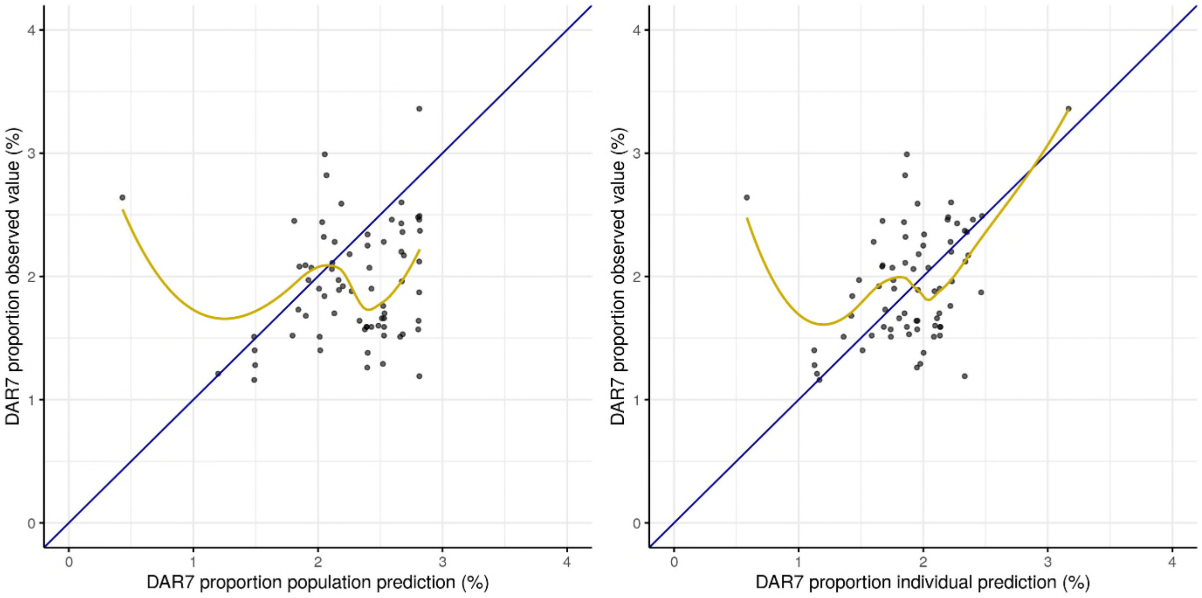

Supplement: Supplementary file 3 — Supplementary material 3 GOF plots, observed data vs population and individual prediction (PDF 1129.8 kb) [file 10928_2021_9799_MOESM3_ESM.pdf]
