# Supplementary material for: Integrated multiple analytes and semi-mechanistic population pharmacokinetic model of tusamitamab ravtansine, a DM4 anti-CEACAM5 antibody-drug conjugate
Source: J Pharmacokinet Pharmacodyn. 2022 Feb 15;49(3):381–94. doi: 10.1007/s10928-021-09799-0 (PMC9098589; doi:10.1007/s10928-021-09799-0)
Supplement: Supplementary file 4 — Supplementary material 4 GOF plots, PWRES, iWRES and NPDE vs time and predicted concentrations (PDF 845.7 kb) [file 10928_2021_9799_MOESM4_ESM.pdf]

Integrated multiple analytes and semi-mechanistic population pharmacokinetic model of tusamitamab ravtansine, a DM4 anti-CEACAM5 antibody-drug conjugate

Journal of Pharmacokinetics and Pharmacodynamics

Clemence Pouzin, Leonid Gibiansky, Nathalie Fagniez, Michel Tod, Mustapha Chadjaa, Laurent Nguyen

Corresponding author: Clemence Pouzin, 1 Avenue Pierre Brossolette, 91380 Chilly-Mazarin, France. Email: Clemence.Pouzin@sanofi.fr

Online Resource 4: GOF plots, PWRES, iWRES and NPDE vs time and predicted concentrations

a. SAR408701

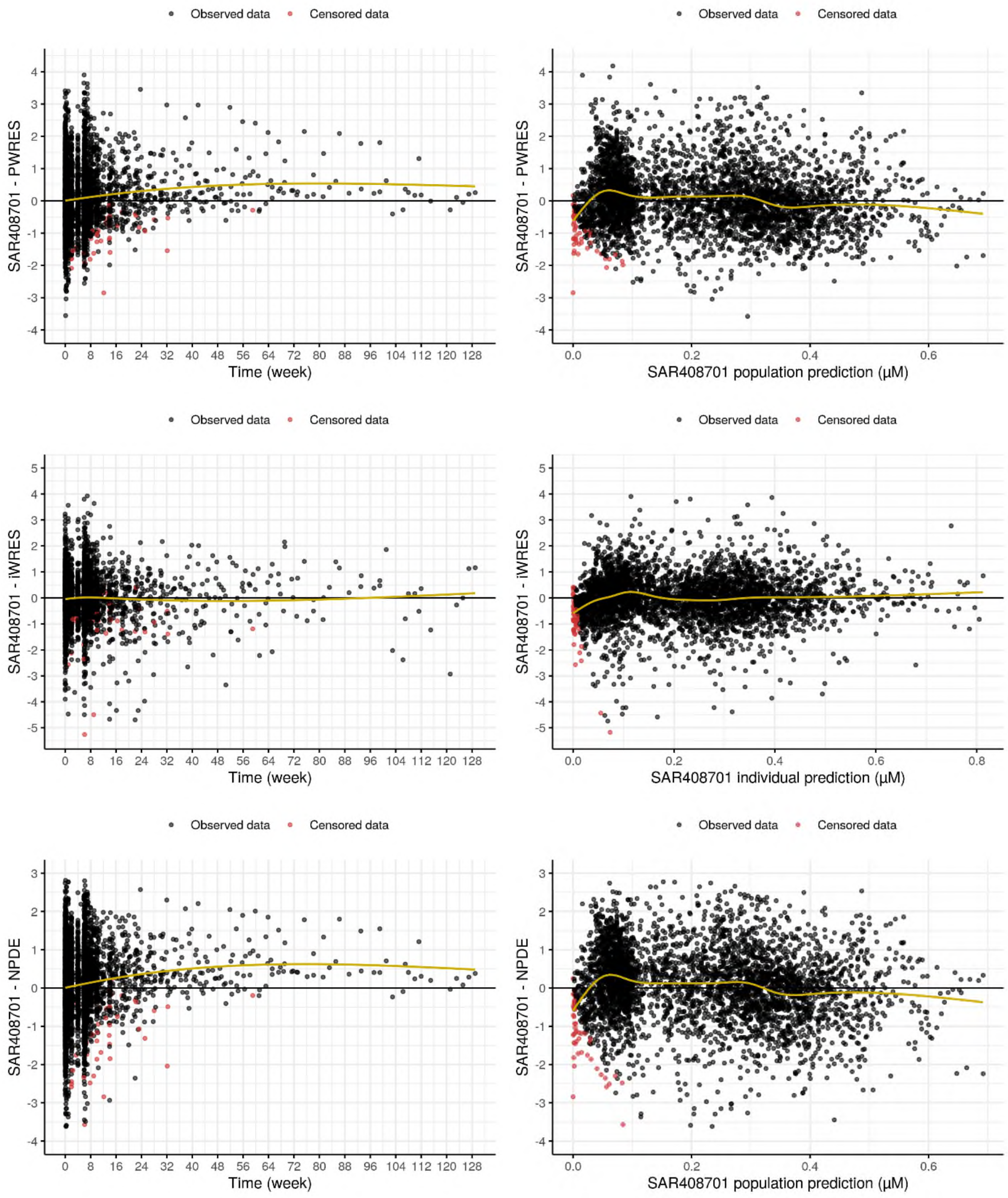

b. DM4

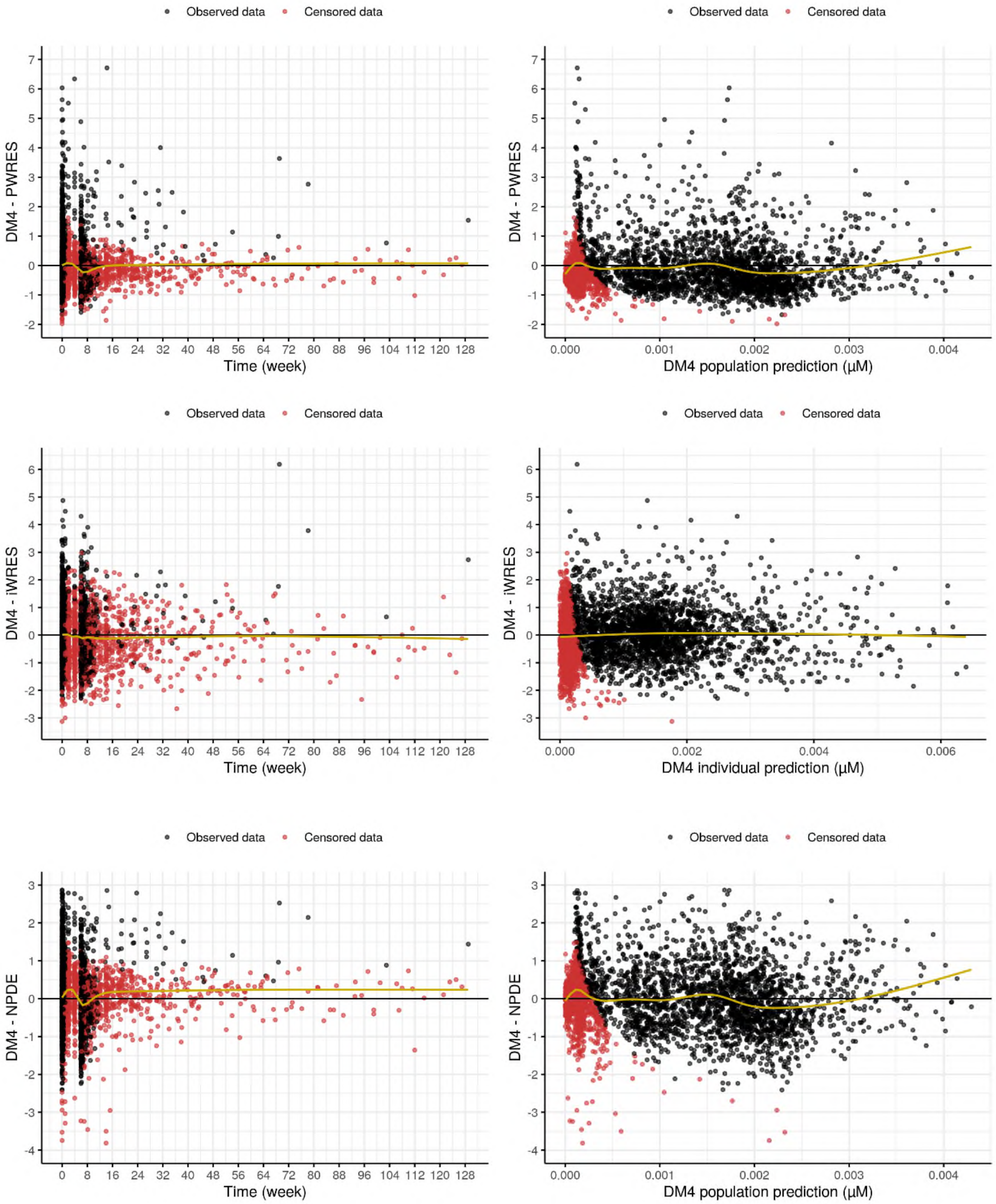

Observed data
Censored data
DM4 - PWRES
Time (week)
DM4 population prediction (µM)
DM4 - iWRES
DM4 individual prediction (µM)
DM4 - NPDE

c. MeDM4

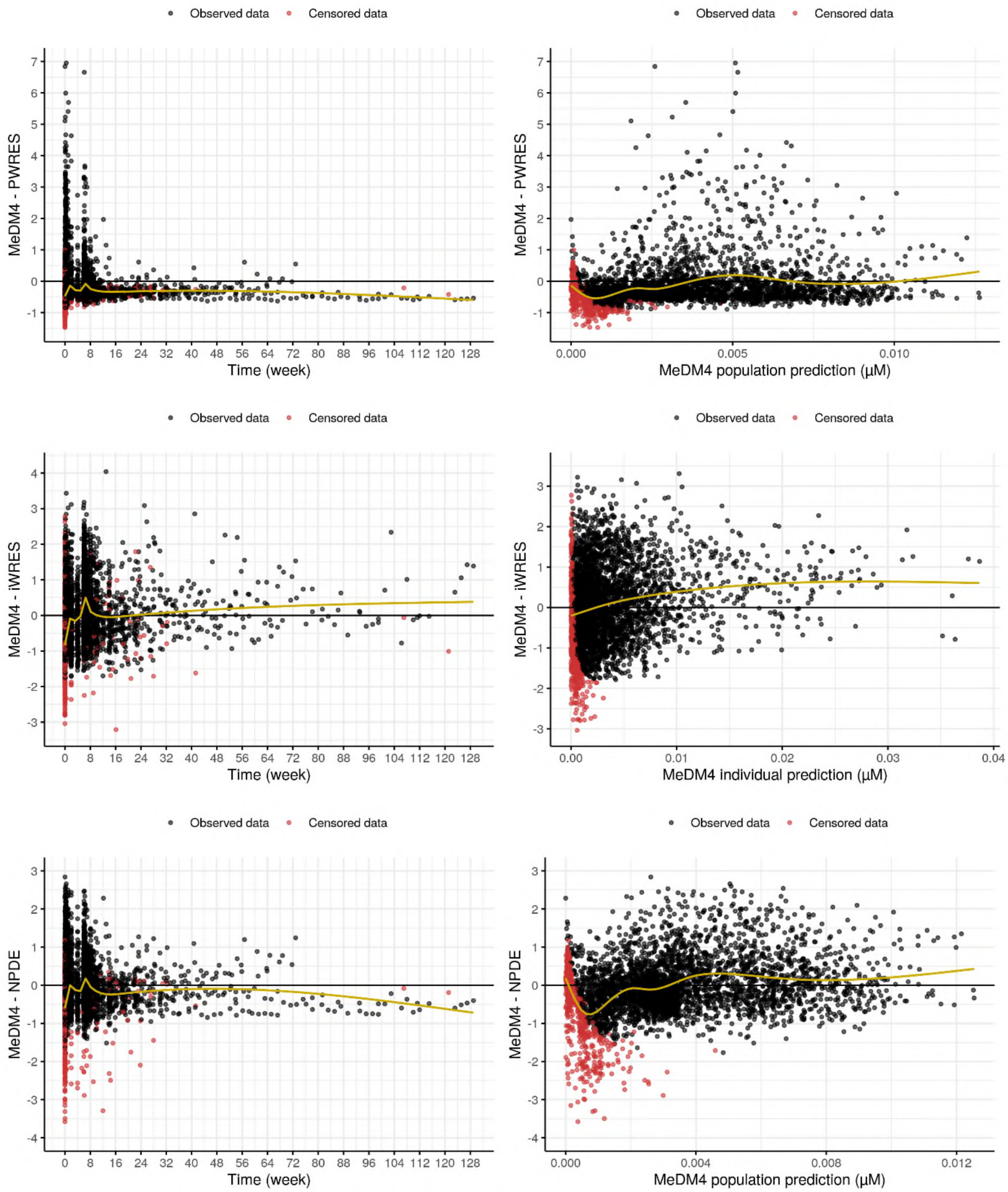

Observed data
Censored data
MeDM4 - PWRES
Time (week)
MeDM4 population prediction (µM)
MeDM4 - iWRES
MeDM4 individual prediction (µM)
MeDM4 - NPDE

d. NAB

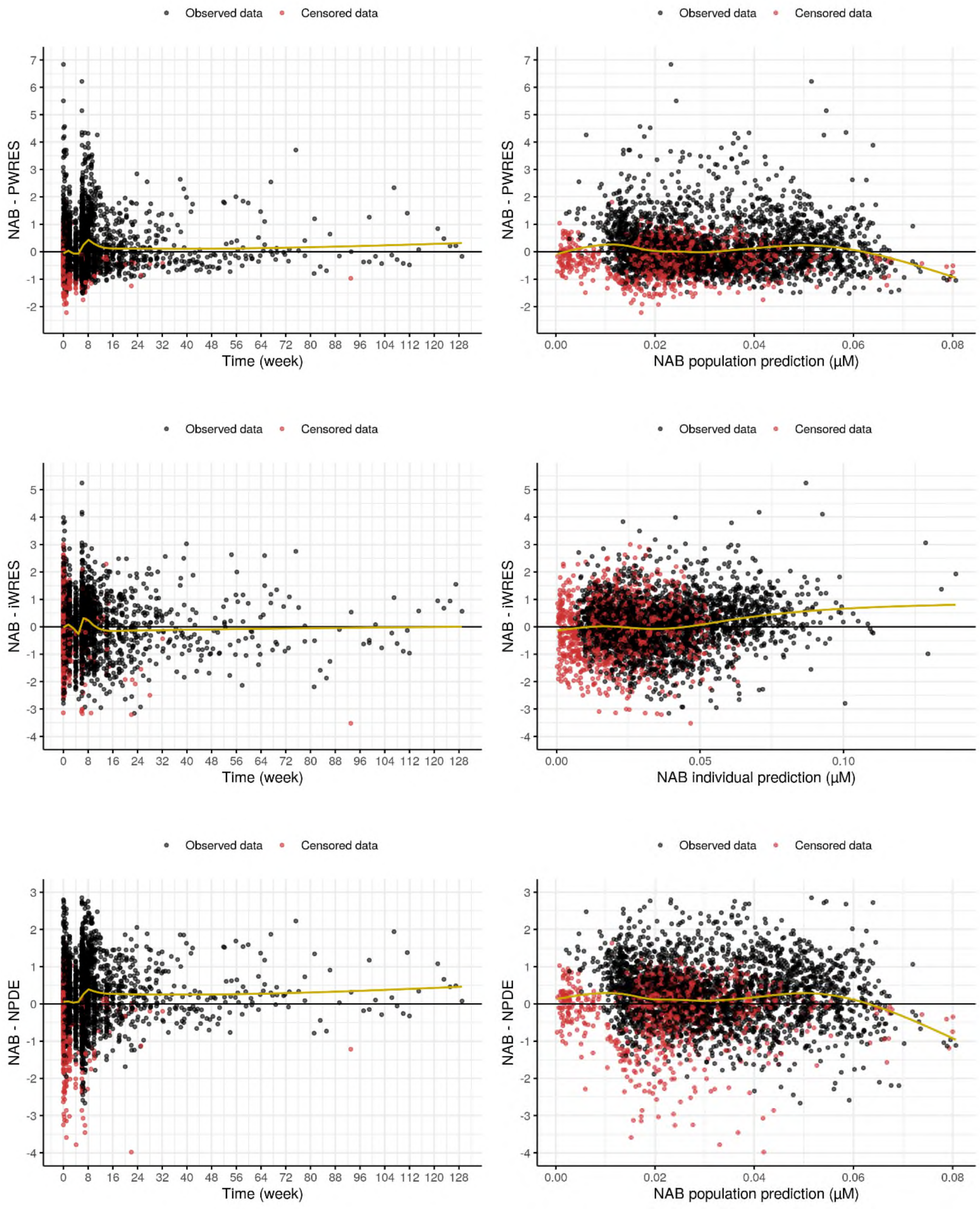

Observed data
Censored data
NAB - PWRES
Time (week)
NAB population prediction (μM)
NAB - iWRES
NAB individual prediction (μM)
NAB - NPDE
