# Supplementary material for: Integrated multiple analytes and semi-mechanistic population pharmacokinetic model of tusamitamab ravtansine, a DM4 anti-CEACAM5 antibody-drug conjugate
Source: J Pharmacokinet Pharmacodyn. 2022 Feb 15;49(3):381–94. doi: 10.1007/s10928-021-09799-0 (PMC9098589; doi:10.1007/s10928-021-09799-0)
Supplement: Supplementary file 5 — Supplementary material 5 pc-VPC at cycle 1 and at cycle 4 (PDF 460.7 kb) [file 10928_2021_9799_MOESM5_ESM.pdf]

Integrated multiple analytes and semi-mechanistic population pharmacokinetic model of tusamitamab ravtansine, a DM4 anti-CEACAM5 antibody-drug conjugate

Journal of Pharmacokinetics and Pharmacodynamics

Clemence Pouzin, Leonid Gibiansky, Nathalie Fagniez, Michel Tod, Mustapha Chadjaa, Laurent Nguyen

Corresponding author: Clemence Pouzin, 1 Avenue Pierre Brossolette, 91380 Chilly-Mazarin, France. Email: Clemence.Pouzin@sanofi.fr

Online Resource 5: pc-VPC at cycle 1 and at cycle 4

a. DM4

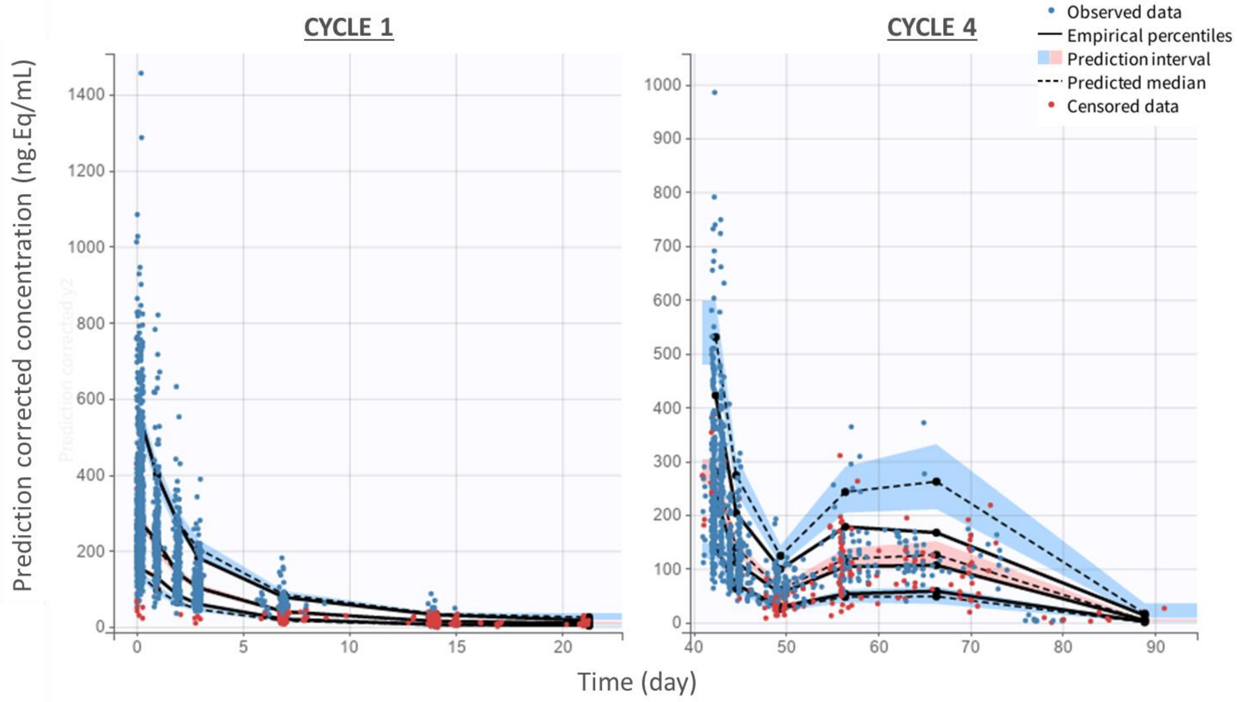

The points represent the observed concentrations (in red the BLQ values, handled as censored data), the solid lines represent the median, 10th and 90th percentiles of the observed data and the blue and red areas represent the prediction intervals for each percentile (at a level of 90%). Concentrations are expressed as SAR408701 equivalent ($Concentration_{ng.Eq/mL} = Concentration_{ng/mL} \times \frac{Molecular\ Weight_{SAR408701}}{Molecular\ Weight_{DM4}}$). The shape of PK profiles may differ at cycle 4 because of dose delay that can be found across cycles for some patients.

b. MeDM4

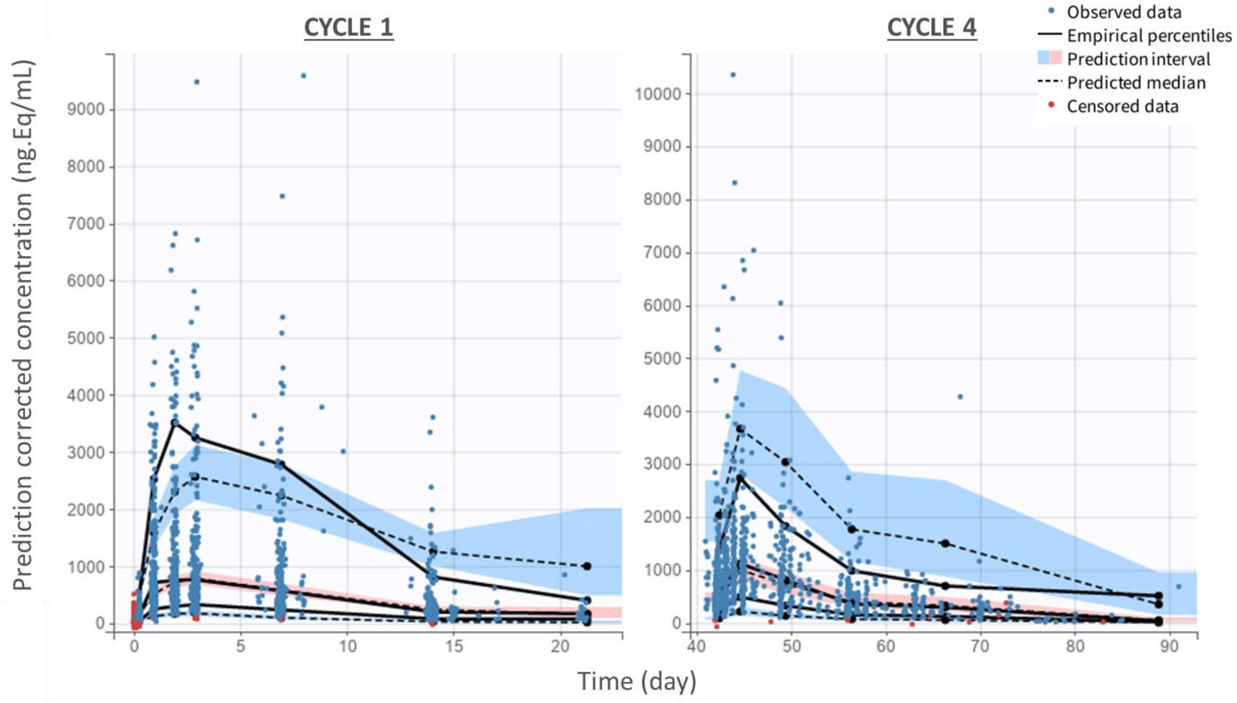

The points represent the observed concentrations (in red the BLQ values, handled as censored data), the solid lines represent the median, 10th and 90th percentiles of the observed data and the blue and red areas represent the prediction intervals for each percentile (at a level of 90%). Concentrations are expressed as SAR408701 equivalent ($Concentration_{ng.Eq/mL} = Concentration_{ng/mL} \times \frac{Molecular\ Weight_{SAR408701}}{Molecular\ Weight_{MeDM4}}$). The shape of PK profiles may differ at cycle 4 because of dose delay that can be found across cycles for some patients.

c. NAB

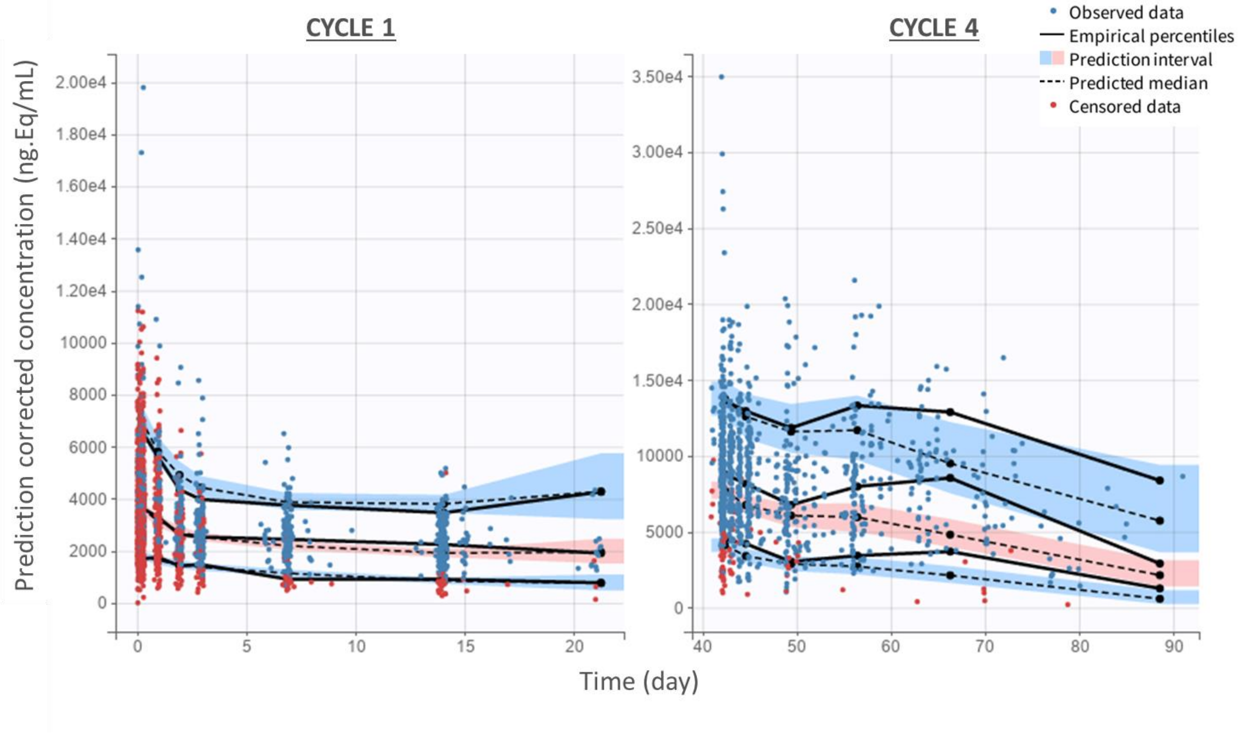

The points represent the observed concentrations (in red the BLQ values, handled as censored data), the solid lines represent the median, 10th and 90th percentiles of the observed data and the blue and red areas represent the prediction intervals for each percentile (at a level of 90%). Concentrations are expressed as SAR408701 equivalent ($Concentration_{ng.Eq/mL} = Concentration_{ng/mL} \times \frac{Molecular\ Weight_{SAR408701}}{Molecular\ Weight_{NAB}}$). The shape of PK profiles may differ at cycle 4 because of dose delay that can be found across cycles for some patients.
